# Distinct H_2_O_2_-Scavenging System in *Yersinia pseudotuberculosis*: KatG and AhpC Act Together to Scavenge Endogenous Hydrogen Peroxide

**DOI:** 10.3389/fmicb.2021.626874

**Published:** 2021-05-07

**Authors:** Fen Wan, Xue Feng, Jianhua Yin, Haichun Gao

**Affiliations:** ^1^College of Laboratory Medicine, Hangzhou Medical College, Hangzhou, China; ^2^Institute of Microbiology and College of Life Sciences, Zhejiang University, Hangzhou, China; ^3^College of Biotechnology and Bioengineering, Zhenjiang University of Technology, Hangzhou, China

**Keywords:** *Yersinia*, OxyR, AhpC, catalase, oxidative stresss response

## Abstract

To colonize in the digestive tract of animals and humans, *Yersinia pseudotuberculosis* has to deal with reactive oxygen species (ROS) produced by host cells and microbiota. However, an understanding of the ROS-scavenging systems and their regulation in this bacterium remains largely elusive. In this study, we identified OxyR as the master transcriptional regulator mediating cellular responses to hydrogen peroxide (H_2_O_2_) in *Y. pseudotuberculosis* through genomics and transcriptomics analyses. OxyR activates transcription of diverse genes, especially the core members of its regulon, including those encoding catalases, peroxidases, and thiol reductases. The data also suggest that sulfur species and manganese may play a particular role in the oxidative stress response of *Y. pseudotuberculosis*. Among the three H_2_O_2_-scavenging systems in *Y. pseudotuberculosis*, catalase/peroxidase KatE functions as the primary scavenger for high levels of H_2_O_2_; NADH peroxidase *a*lkyl *h*ydro*p*eroxide *r*eductase (AhpR) and catalase KatG together are responsible for removing low levels of H_2_O_2_. The simultaneous loss of both AhpC (the peroxidatic component of AhpR) and KatG results in activation of OxyR. Moreover, we found that AhpC, unlike its well-characterized *Escherichia coli* counterpart, has little effect on protecting cells against toxicity of organic peroxides. These findings provide not only novel insights into the structural and functional diversity of bacterial H_2_O_2_-scavenging systems but also a basic understanding of how *Y. pseudotuberculosis* copes with oxidative stress.

## Introduction

Oxidative stress caused by reactive oxygen species (ROS), including superoxide (O_2_^–^), hydrogen peroxide (H_2_O_2_), and hydroxyl radical (OH^⋅^), is inevitable to all organisms that respire oxygen (Li et al., 2016). These strong oxidants and/or radicals could damage virtually all biomolecules, such as nucleic acids, proteins, and lipids (Imlay, 2013). Naturally, detoxification of ROS is extremely critical for survival of diverse bacteria, pathogens in particular, because the host cells release ROS as a deadly weapon to defend against bacterial infections (Fang, 2004). Among ROS, H_2_O_2_ not only can be formed very rapidly endogenously (for example, 15 μM s^–1^ in *Escherichia coli*) but also enters into cells nearly freely, where it reacts with Fe^2+^ to generate most deadly hydroxyl radicals (Li et al., 2016). Because of these features, most bacteria employ multiple enzymes, including catalases, various peroxidases, and rubrerythrin, to keep the intracellular concentrations of H_2_O_2_ at safe limit (nanomolar levels; Mishra and Imlay, 2012).

Catalases are principal enzymes that protect bacterial cells against H_2_O_2_ stress by catalyzing H_2_O_2_ into water and oxygen (Mishra and Imlay, 2012). Two types of catalases are present in bacteria: one is bifunctional (or called catalase-peroxidase, HPI, e.g., *E. coli* KatG), with both catalytic and peroxidatic activities, and the other is monofunctional (HPII, e.g., *E. coli* KatE), with only catalytic activity (Mishra and Imlay, 2012). Although it is well recognized that catalases serve as the primary scavenger of H_2_O_2_, they generally work best with H_2_O_2_ at high levels (millimolar levels; Mishra and Imlay, 2012). One of the best characterized peroxidases is NADH peroxidase AhpR (named from *a*lkyl *h*ydro*p*eroxide *r*eductase), which could decompose both H_2_O_2_ and organic peroxides (OPs) in diverse bacteria (Jacobson et al., 1989; Niimura et al., 1995). Compared to catalase, AhpR is more efficient in scavenging low-level H_2_O_2_ and regarded as the primary scavenger of endogenous H_2_O_2_ (Seaver and Imlay, 2001). AhpR typically consists of two cytoplasmic proteins encoded by a single operon, the peroxidase component AhpC and its cognate reductase AhpF: the former is a bacterial representative of typical 2-Cys peroxiredoxins and the latter is a flavoprotein with NADH:disulfide oxidoreductase activity (Poole, 2005). AhpC reduces H_2_O_2_ by oxidizing the two conservative cysteines to form an intermolecular disulfide bond, which is reactivated by AhpF using NADH as reducing equivalent (Perkins et al., 2015). Atypical AhpR systems, in which AhpF is absent, have been found in some bacteria (Bryk et al., 2000; Baker et al., 2001). For instance, in *Helicobacter pylori*, AhpC is reduced by a thioredoxin/thioredoxin reductase (TrxB) system, and in *Mycobacterium tuberculosis*, AhpD reduces AhpC with electrons from NADH through dihydrolipoamide dehydrogenase and dihydrolipoamide succinyltransferase (Baker et al., 2001; Jaeger et al., 2004). Because of the essential roles of catalase and AhpR in H_2_O_2_-scavenging, bacterial strains lacking both together display a drastically elevated sensitivity to H_2_O_2_ and carry an apparent aerobic growth defect (Cosgrove et al., 2007; Ezraty et al., 2017).

In many bacteria, OxyR is a primary transcriptional regulator that mediates cellular response to oxidative stress (Imlay, 2013; Fu et al., 2015). As a LysR-family DNA-binding protein, OxyR senses and responds to H_2_O_2_ stress *via* formation of an intramolecular disulfide bond between two conserved cysteine residues [Cys 199 and Cys 208 within *E. coli* OxyR (*Ec*OxyR); Zheng et al., 1998]. The regulatory mode of OxyR varies among bacteria. For example, in *E. coli*, *Salmonella enterica* serovar Typhimurium (*S*. Typhi), and many other bacteria, OxyR functions as an activator only for major H_2_O_2_-scavenging proteins, such as catalases and AhpR (Imlay, 2008). However, it can also function in a dual-control manner (both a repressor and an activator for certain genes) in *Neisseria* and *Shewanella oneidensis* or as a repressor only in *Corynebacterium diphtheriae* (Ieva et al., 2008; Kim and Holmes, 2012; Jiang et al., 2014). OxyR proteins now have been generally regarded as a global regulator implicated in diverse biological processes, but the core members of their regulons are consistently constituted by operons responsible for H_2_O_2_ degradation such as catalases and peroxidases, iron-sequestering proteins, and thioredoxin and glutathione antioxidant systems (Imlay, 2015).

*Yersinia pseudotuberculosis* is a Gram-negative enteric pathogen that often causes self-limiting gastrointestinal disorders such as enteritis, diarrhea, and mesenteric lymphadenitis to animals and humans (Brady et al., 2020). During infection, environmental stress and host immunity reactions in human guts can introduce an increase in levels of ROS that *Y. pseudotuberculosis* cells encounter, thus requiring a sophisticated regulation of genes to reduce the intracellular H_2_O_2_ level to a safe line (Knaus et al., 2017). It has been reported that *Y. pseudotuberculosis* is able to combat oxidative stress by an unconventional way: it imports zinc to mitigate ROS by secreting a zincophore *via* the type VI secretion system (T6SS; Wang et al., 2016, 2020). However, how this bacterium copes with oxidative stress has not been systematically investigated and thus is poorly understood.

In this study, we carried out the transcriptomics analysis of *Y. pseudotuberculosis* YPIII in response to exogenous H_2_O_2_. We identified an OxyR analog in YPIII and found that its regulon members are highly conserved, including those encoding H_2_O_2_-scavenging enzymes, iron-sequestering proteins, and thiol-reducing systems. Our data support that OxyR of YPIII functions in an activator-only mode, and its absence causes a plating defect on LB agar, a result of insufficient production of H_2_O_2_-scavenging enzymes, including KatE, KatG, and AhpC. We further showed that KatE functions as the primary scavenger for H_2_O_2_, but both KatG and AhpC together play an essential role in decomposing low levels of H_2_O_2_. The simultaneous loss of both KatG and AhpC results in activation of OxyR, which in turn upregulates KatE production, conferring cells’ enhanced resistance to H_2_O_2_. The findings presented here reveal a novel mechanism through which bacterial cells differentially exploit individual components of the H_2_O_2_-scavenging repertoire to increase fitness in harsh living environments.

## Materials and Methods

### Bacterial Strains, Plasmids, and Culture Conditions

All bacterial strains and plasmids used in this study are listed in Table 1. All chemicals were obtained from Sigma (Shanghai, China) unless otherwise noted. For genetic manipulation, *E. coli* and *Y. pseudotuberculosis* were grown in LB (containing 1% tryptone, 0.5% yeast extract, and 0.5% NaCl) under aerobic conditions at 37 and 26°C. When needed, the following chemicals were added to the growth medium: 2,6-diaminopimelic acid (DAP), 0.3 mM; ampicillin, 50 μg/ml; kanamycin, 50 μg/ml; gentamycin, 15 μg/ml; and streptomycin, 100 μg/ml.

**TABLE 1:** Bacterial strains and plasmids used in this study.

Strain or plasmid	Description	Source or references
**Strains**		
*E. coli*		
DH5α	Host strain for cloning	Lab stock
WM3064	ΔdapA, donor strain for conjugation	W. Metcalf, UIUC
*S. oneidensis*		
MR-1	Wild type	
HG1070	ΔkatB derived from MR-1	Jiang et al., 2014
HG0958-1070	ΔkatBΔahpC derived from MR-1	Feng et al., 2020
***Y. pseudotuberculosis***		
YPIII	Wild type	Rosqvist et al., 1988
YPK_RS20585	ΔoxyR derived from YPIII	This study
YPK_RS14285	ΔkatE derived from YPIII	This study
YPK_RS17025	ΔkatG derived from YPIII	This study
YPK_RS16370	ΔahpC derived from YPIII	This study
YPK17025-16370	ΔkatGΔahpC derived from YPIII	This study
YPK14285-17025	ΔkatEΔkatG derived from YPIII	This study
YPK3CAT	ΔkatEΔkatGΔahpC derived from YPIII	This study
**Plasmids**		
pHGM01	Ap^r^Gm^r^Cm^r^,att-based suicide vector	Jin et al., 2013
pHG101	Promoterless vector for complentation	Wu et al., 2011
pHGEI01	Km^r^, integrative lacZ reporter vector	Fu et al., 2014
pHGEI01-katE	pHGEI01 containing the katE promoter	This study
pHGEI01-katG	pHGEI01 containing the katG promoter	This study
pHGEI01-ahpC	pHGEI01 containing the ahpC promoter	This study

### Transcriptomics Analysis

For transcriptomics analysis, cell samples were prepared as described previously (Jiang et al., 2014). In brief, cultures of the mid-exponential phase (∼0.4 of OD_600_, the same throughout the study) were subjected to the H_2_O_2_ treatment. H_2_O_2_ was added to a final concentration of 0.5 mM, and cells were collected before and 5 min after the addition. Cells were pelletted at 14,000 rpm for 30 s at room temperature and frozen immediately in liquid nitrogen. Three biological replicates under each condition were prepared. Total RNA was extracted with RNeasy Mini Kit (QIAGEN, Alameda, United States), and RNA sequencing (RNA-seq) analysis was conducted as before (Gao et al., 2004; Sun et al., 2020). Data analysis was conducted according to standard procedures used previously (Sun et al., 2020). Genes that passed statistical analysis by analysis of variance (ANOVA; *p* < 0.05) with Benjamini–Hochberg false discovery rate multiple-testing correction and showed two-fold difference between the H_2_O_2_-treated and untreated control samples were discussed in the study. NCBI SRA accession number is PRJNA671546 for raw transcriptomics analysis data.

### Real-Time Quantitative RT-PCR

Quantitative reverse transcription-PCR (qRT-PCR) was performed to verify the expression of key OxyR regulon members with an ABI7300 96-well qRT-PCR system (Applied Biosystems) as described previously (Jiang et al., 2014). The expression of each gene was determined from four replicas in a single real-time qRT-PCR experiment. The cycle threshold (*C*_*T*_) values for each gene of interest were averaged and normalized against the *C*_*T*_ value of the 16s rRNA gene, whose abundance was constant during the exponential phase. Relative abundance (RA) of each gene was standardized to the *C*_*T*_ values of the 16s rRNA gene using the equation RA = 2^–Δ^*^*CT*^*.

### Mutagenesis, Complementation of Mutant Strains

Yersinia *pseudotuberculosis* in-frame deletion strains were constructed by the *att*-based Fusion PCR method as described previously (Jin et al., 2013). In brief, two fragments flanking the target gene were generated by PCR with primers containing *attB* and the gene-specific sequence, which were linked by a linker sequence *via* second round of PCR. The fusion fragments were integrated into plasmid pHGM01 by site-specific recombination using Gateway BP clonase II enzyme mix (Invitrogen). The resulting vectors were introduced in *E. coli* WM3064 and transferred to *Y. pseudotuberculosis* by conjugation. Integration of the mutagenesis constructs into the chromosome was selected by resistance to gentamycin and confirmed by PCR. Verified *trans*-conjugants were grown in LB broth without NaCl and plated on LB supplemented with 10% sucrose. Gentamycin-sensitive and sucrose-resistant colonies were screened by PCR for deletion of the target gene. To facilitate growth of mutants, catalase (from bovine liver, Sigma) was added onto the plates. All mutations were verified by sequencing the mutated regions.

Plasmid pHG-101 was used in genetic complementation of mutants as described before (Wu et al., 2011). For complementation of genes next to their promoter, a fragment containing the gene of interest and its native promoter was amplified by PCR and cloned into pHG-101. After sequencing verification, the resulting vectors were transferred into the relevant strains *via* conjugation.

### Growth and Susceptibility to H_2_O_2_ or *t-*BHP

The spotting assay was used to evaluate the plating defect on LB plates. Cells of the mid-exponential phase were collected by centrifugation and adjusted to 10^9^ cells/ml, which was set as the undiluted (dilution factor 0). Ten-fold serial dilutions were prepared with fresh LB medium. Five microliter of each dilution was spotted onto LB plates. The plates were incubated for 24 h or longer in the dark before being photographed. All experiments were repeated at least three times.

Disk diffusion assays to test for sensitivity to oxidative stress conditions were performed with *Y. pseudotuberculosis* strains. Two hundred microliter of mid-exponential phase cultures were spread onto LB plates, 6 mm (in diameter) paper disks loaded with 10 μl H_2_O_2_ or *tert*-butyl hydroperoxide (*t-*BHP) of various concentrations were placed onto the bacterial lawn grown for 6 h, and plates were incubated 26°C for 16 h.

### H_2_O_2_ Quantification

H_2_O_2_ at high concentrations (>100 μM) was quantified using the ferrous ion oxidation-xylenol orange method (Wolff, 1994). In brief, cells of the mid-exponential phase grown in liquid LB were collected by centrifugation, washed in PBS, and resuspended in the same buffer to an OD_600_ of 0.1. H_2_O_2_ was added to final concentrations indicated in the figure legends. Cells were filtered out at different time points, and elutions were assayed immediately for the remaining H_2_O_2_.

H_2_O_2_ at low concentrations (<50 μM) was quantified using Amplex red fluorescent method (Seaver and Imlay, 2001). In the presence of H_2_O_2_, Amplex red can be oxidized by horseradish peroxidase to the fluorescent product resorufin. To measure H_2_O_2_, 200 μl of samples from cells grown in MS medium was mixed with 100 μl of stock solutions for Amplex red and horseradish peroxidase prepared the same as described elsewhere (Seaver and Imlay, 2001). Fluorescence was then measured in a Synergy 2 Pro200 Multi-Detection Microplate Reader (Tecan) and converted to H_2_O_2_ concentration using a curve obtained from standard samples.

### β-Galactosidase Activity Assay

β-Galactosidase activity assay was used to determine gene expression. The sequence in sufficient length (∼400 bp) upstream of gene of interest was amplified and inserted in front of the full-length *E. coli lacZ* gene in plasmid pHGEI01 (Fu et al., 2014). The resulting plasmid was verified by sequencing, introduced into *E. coli* WM3064, and then conjugated with relevant *Y. pseudotuberculosis* strains. Cultures of the mid-exponential phase were collected by centrifugation, washed with PBS, and treated with lysis buffer (0.25 M Tris/HCl, 0.5% Triton X-100, and pH 7.5). Extracts were collected by centrifugation and applied for enzyme assay by adding *o*-nitrophenyl-β-*D*-galactopyranoside (4 mg/ml). Changes in absorption over time were monitored at 420 nM with a Synergy 2 Pro200 Multi-Detection Microplate Reader (Tecan), and the results were presented as Miller units.

### Bioinformatics and Statistical Analyses

Multiple sequence alignment was carried out with Clustal Omega (Madeira et al., 2019). Sequence logos were generated by using WebLogo (Crooks et al., 2004). Three-dimensional structures of YpAhpC were predicted using Phyre^1^ (Kelley et al., 2015). The predicted structures were then visualized by software Pymol (DeLano Scientific LLC). Genome screening for OxyR-binding sites based on established weight matrixes from various bacteria was performed using regulatory sequence analysis tools (RSATs; Medina-Rivera et al., 2015). For statistical analysis, Student’s *t*-test was performed for pairwise comparisons of groups, and values are presented as means ± standard deviation (SD).

## Results

### Genomics Analysis of *Y. pseudotuberculosis* YPIII With Respect to Oxidative Stress Response

To identify the oxidative stress response regulator(s) in YPIII, a BLASTp search of functional analogs of established oxidative stress-responding regulators, including *Ec*OxyR and SoxRS as well as *Bacillus subtilis* PerR, against the YPIII proteome was performed. While no homolog of *E. coli* SoxRS or of *B. subtilis* PerR was found, YPIII possesses a highly confident homolog of *Ec*OxyR, YPK_RS20585 (BLASTp *E*-value = 0; identities, 88%; Supplementary Figure 1). Like all 2-Cys OxyRs, YPK_RS20585 contains two conserved cysteine residues (Cys199 and Cys208 within both YPK_RS20585 and *Ec*OxyR) implicated in the activation by disulfide bond formation, suggesting a possible role in the oxidative stress response of YPIII, and therefore we named it OxyR (*Yp*OxyR). However, the sequence similarities between *Yp*OxyR and dual-activity OxyRs, such as that of *S. oneidensis*, are substantially lower (*E*-value = 7e-47; identities, 33%; Supplementary Figure 2), implying that *Yp*OxyR might function as an activator only.

The current knowledge on bacterial oxidative stress response is most well developed in *E. coli*, whose OxyR regulon is composed of over 20 operons (Imlay, 2015). The most important and conserved *Ec*OxyR regulon members encode proteins involved in detoxification and prevention and/or repair of oxidative damage, such as catalases and peroxidases, iron-sequestering proteins, thioredoxin, and glutathione antioxidant systems. Similarly, the YPIII genome encodes two catalases (HPII KatE and HPI KatG), iron-sequestering protein Dps, and a complete set of thioredoxin and glutathione antioxidant proteins, including TrxA (thioredoxin), TrxB (thioredoxin-disulfide reductase), TrxC (thioredoxin), GrxA (glutaredoxin), YPK_RS20590 (glutathione peroxidase), and GorA (glutathione-disulfide reductase; Table 2).

**TABLE 2:** Highly regulated genes involved in the response to H_2_O_2_ and genes encoding analogs of OxyR regulon members.

Locus tag	Gene	OxyR regulon^a^	YpOxyR motif W^b^	Fold Change^c^	COG type^d^	Description
**Top 20 up-regulated genes**						
YPK_RS20590		Y		689.5	O	Glutathione peroxidase
YPK_RS20595				89.3		Dihydrolipoyl dehydrogenase
YPK_RS16760	*trxC*	Y		75.4	J	Thioredoxin TrxC
YPK_RS08095	*dps*	Y	15.4	48.3	P	Iron-sequestering protein Dps
YPK_RS17250	*cysC*			38.5	P	Adenylyl-sulfate kinase
YPK_RS22535				34.0		Hypothetical protein
YPK_RS17260	*cysD*			32.0		Sulfate adenylyltransferase subunit CysD
YPK_RS17255	*cysN*			28.3		Sulfate adenylyltransferase subunit CysN
YPK_RS14285	*katE*	Y	22.7	27.6	P	Catalase, OxyR regulon
YPK_RS13675	*grxA*	Y	12.6	22.6	S	GrxA family glutaredoxin
YPK_RS17265	*cobA*			18.0		Uroporphyrinogen-III C-methyltransferase
YPK_RS00550	*gorA*	Y		16.2	C	Glutathione-disulfide reductase
YPK_RS13735				16.2		Isopenicillin N synthase family oxygenase
YPK_RS13730				15.4		ABC transporter substrate-binding protein
YPK_RS03760				13.2		TonB-dependent receptor
YPK_RS09490				13.1		CMD domain-containing protein
YPK_RS07100	*cysA*			13.0		Sulfate/thiosulfate ABC transporter ATP-binding protein CysA
YPK_RS16765				12.7		DTW domain-containing protein
YPK_RS10680	*zwf*			12.3	G	Glucose-6-phosphate dehydrogenase
YPK_RS07190	*cysK*			11.9	G	Cysteine synthase A
**Other OxyR regulon members**						
YPK_RS17025	*katG*	Y	23.1	7.2	P	Catalase/peroxidase HPI
YPK_RS20585	*oxyR*	Y		4.5		DNA-binding transcriptional regulator OxyR
YPK_RS13445	*trxB*	Y	21.0	4.5	O	Thioredoxin-disulfide reductase
YPK_RS16370	*ahpC*	Y	21.1	3.5	O	Peroxiredoxin C
YPK_RS09285	*sufA*	Y		2.598	P	Fe-S cluster assembly protein

*^*a*^Y, OxyR regulon members in at least three bacterial genera (*E. coli*, *S. Typhi*, and *Neisseria meningitidis*). ^*b*^Weight values of predicted *Yp*OxyR regulon members by using regulatory sequence analysis tool (RSAT). ^*c*^Fold changes in transcripts (the ratio of the H_2_O_2_-treated sample of the wild type to the control) revealed in transcriptomics analysis. ^*d*^COG: O: posttranslational modification; J: translation, ribosomal and biogenesis; P: metabolism; S: Function unknown; C: energy production; and G: carbohydrate transport and metabolism.*

Perhaps one of the most striking observations is that YPIII seemingly lacks the counterpart of *E. coli* AhpF, the cognate reductase for peroxidase component AhpC of AhpR (Tartaglia et al., 1990; Poole and Ellis, 1996). A BLASTp search using *E. coli* AhpCF against the YPIII proteome revealed a single putative homolog for AhpC and AhpF, YPK_RS16370 (peroxiredoxin C; *E*-value, 3e-38) and TrxB (*E*-value, 3e-42), which are not in proximity on the chromosome. Combining the fact that AhpCF is encoded by a single operon in other bacteria and TrxB is clearly the counterpart of *Ec*TrxB (*E*-value, 0) but not *Ec*AhpF, these data strongly suggest that YPIII lacks a conventional AhpF. Furthermore, the YPIII genome also lacks a gene for a homolog of Ohr, the primary enzyme that decomposes OPs. Given the involvement of AhpR in scavenging OPs, thus it is particularly important and interesting to understand the physiological role of AhpC in YPIII.

### Transcriptomics Analysis of YPIII in Response to H_2_O_2_ Stress

**FIGURE 1:**
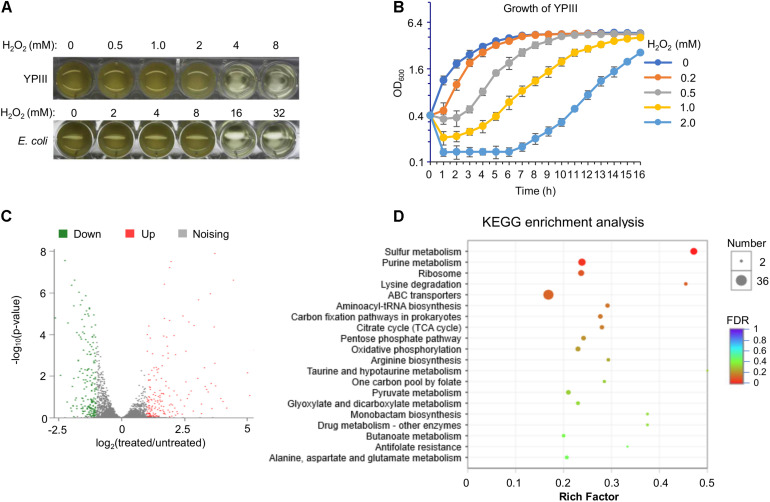
Characteristics of *Y. pseudotuberculosis* in response to H_2_O_2_. **(A)** Minimum inhibitory concentration (MIC) assay. Mid-exponential phase cultures (OD_600_ of ∼0.4) were used to inoculate each well to an OD_600_ of 0.01, and MIC was determined 16 h later. **(B)** Impact of H_2_O_2_ on growing cells. H_2_O_2_ was added to mid-exponential phase cultures of the wild type to the final concentrations as indicated. Growth was monitored by recording OD_600_ values. **(C)** Volcano plot of the different expression genes in wild-type cells between before and after the H_2_O_2_ treatment (0.5 mM). **(D)** Kyoto Encyclopedia of Genes and Genomes (KEGG) pathway enrichment analysis of differentially expressed genes in wild-type cells between before and after the H_2_O_2_ treatment.

In order to gain a comprehensive understanding of the cellular response of YPIII to H_2_O_2_, we performed an RNA-seq analysis to obtain gene expression changes resulting from the H_2_O_2_ treatment. Our early stress response studies suggest that the best concentration of H_2_O_2_ for transcriptomics analyses would be the dosage at which the agent arrests growth of cells but does show an evident killing effect on them (Gao et al., 2004; Jiang et al., 2014). To determine the concentration, the minimum inhibitory concentration (MIC) of H_2_O_2_ against the wild-type strain of YPIII was assessed. The results revealed MIC to be 4 mM, which is four times lower than that of *E. coli* (Figure 1A), indicating that YPIII is significantly more sensitive to H_2_O_2_ than *E. coli*. Then, impacts of H_2_O_2_ over a range of concentrations (under MIC) on growth and viability of actively growing cells (∼0.4 of OD_600_) were examined. Upon addition of H_2_O_2_ at 0.2 mM or higher, growth paused immediately and resumed after lag periods that increase with the H_2_O_2_ concentrations (Figure 1B). Additionally, viability assays revealed that H_2_O_2_ at 0.5 mM or lower did not show a significant killing effect (Supplementary Figure 3). Based on these observations, for RNA-seq transcriptomics analysis, we collected cells before and 5 min after the addition of 0.5 mM H_2_O_2_ as the untreated control and the treated samples, respectively.

In total, 364 genes displayed significant differences in transcription levels between the H_2_O_2_-treated and untreated control samples (Supplementary Table 1). Among these genes, 186 genes were up-regulated, while 178 genes were down-regulated (Figure 1C). Genes displaying significant differences in expression levels due to H_2_O_2_-induced oxidative stress were observed in almost every functional category, indicating that YPIII transcriptionally responds to H_2_O_2_ in a global scale (Supplementary Table 1). The high quality of the expression data was validated with a statistical analysis as previously described (Gao et al., 2004) and by real-time qRT-PCR. Eight genes were selected for analysis with the same RNA samples used in the RNA-seq based on the level and reproducibility of changes observed in the RNA-seq experiments. A high level of concordance (*R*^2^ = 0.96) was observed between RNA-seq and real-time qRT-PCR data despite quantitative differences in the level of change (Supplementary Figure 4), suggesting that the RNA-seq results are an accurate reflection of the gene expression profile. The highly induced included many genes encoding proteins combating oxidative stress. In contrast, genes encoding metabolic enzymes were down-regulated in general (Figure 1D), an observation that reflects a paused/reduced growth rate rather than a specific response to the stress has been commonly found in other stress response studies (Gao et al., 2004; Jiang et al., 2014). Intriguingly, many ABC transporters and sulfur metabolic enzymes are among differentially expressed genes, implicating that sulfur species and certain small molecules may have a particular significance in the oxidative stress response of YPIII.

To stay focused, here we only discussed genes whose transcription was significantly induced upon the addition of H_2_O_2_, especially those specific to H_2_O_2_-induced oxidative stress (Table 2). With respect to OxyR regulon members, our transcriptomics data revealed that YPK_RS20590 (glutathione peroxidase), *trxC*, *dps*, *katE*, *grxA*, and *gorA* were among the top 20 most up-regulated genes in H_2_O_2_-treated cells (Table 2). Other putative OxyR regulon members that were induced but not among the top 10 included *katG*, *trxB*, *ahpC*, and *sufABC* (Fe-S cluster assembly proteins; Table 2). Intriguingly, the entire thioredoxin and glutathione antioxidant systems, which play a supporting role in combating oxidative stress in many bacteria (Feng et al., 2019), were found to be highly induced by H_2_O_2_ (for instance, YPK_RS20590 was induced 689-fold), implying that these systems may have more significant contribution in protecting YPIII cells from oxidative damage. It is also worth mentioning that the *oxyR* gene was transcribed at substantially elevated levels (4.5-fold) upon H_2_O_2_ stress (Table 2), suggesting that the regulator *per se* is also subjected to quantity control, in addition to activity transformation upon oxidation.

Among the remaining top 20 most significantly induced genes, those for sulfur species transport and metabolism (*cysC*, *cysD*, *cysN*, *cysA*, and *cysK*) drew special attention. In many organisms, sulfur species are critically involved in cell protection against oxidative damage (Wu et al., 2015; Peng et al., 2017; Korshunov et al., 2020). Along with highly induced transcription of *cysJ*, *cysW*, *cysT*, and *cysI* in H_2_O_2_-challenged cells, these data suggest that sulfur species may be critical for oxidative stress response in YPIII. In addition, we also noticed that genes encoding iron/manganese ABC transport system (*yfeA*, *yfeB*, *yfeC*, and *yfeD*) were transcribed at significantly elevated levels upon the treatment. This may not be surprising given that the intracellular Mn/Fe ratios are correlated with bacterial resistance to oxidative stress (Chandrangsu et al., 2017). To maintain the activity of vulnerable mononuclear iron proteins in the presence of H_2_O_2_, many bacteria replace the iron atom with manganese as the active center (Anjem et al., 2009; Imlay et al., 2019). These data concur well with the result from the Kyoto Encyclopedia of Genes and Genomes (KEGG) enrichment analysis in which sulfur metabolic pathways are among the most enriched (Figure 1D). Together with the missing of AhpF, these results suggest that the mechanism underlying the response of YPIII to H_2_O_2_-induced oxidative stress carries novel characteristics that are different from the classical mode established in *E. coli*.

### Characterization of YPIII *oxyR* Mutant

**FIGURE 2:**
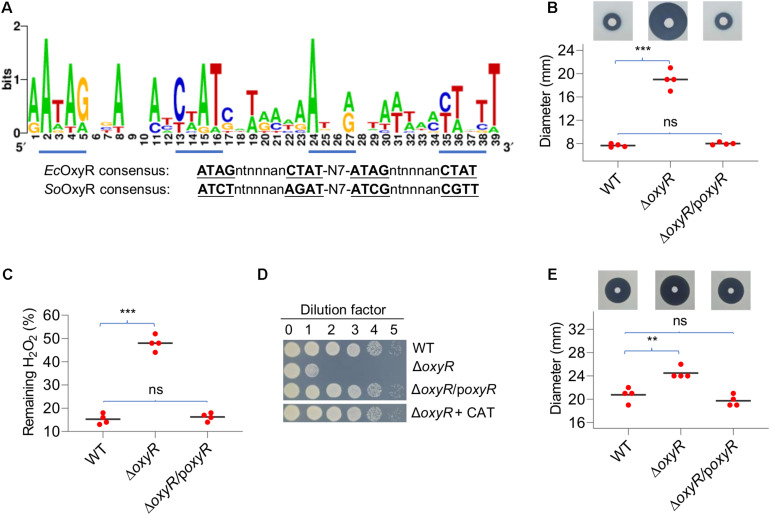
Characteristics of the *Y. pseudotuberculosis oxyR* mutant. **(A)** OxyR-binding motifs in YPIII derived from the top 20 members of the predicted regulon. The matrix for screening the YPIII genome was generated using most conserved members of OxyR regulons of diverse bacteria. Tetranucleotide sequences are underlined based on the *E. coli* and *S. oneidensis* OxyR consensus. **(B)** Disk diffusion assay for H_2_O_2_. Paper disks loaded with 10 μl of 5 M H_2_O_2_ were placed onto bacterial lawns (pregrown for 6 h). Results shown are from 24 h after the disks were placed. The sensitivity was represented by the diameter of the inhibition zone. p*oxyR* represents a copy of *oxyR* to be expressed *in trans* for complementation. **(C)** Measuring H_2_O_2_-scavenging rates. Cells grown to the mid-exponential phase were collected, washed, and suspended to an OD_600_ of ∼0.4. 5 min after the addition of 0.5 mM H_2_O_2_, the remaining H_2_O_2_ was determined. The data were normalized to the initial concentration. **(D)** Plating defects of the *oxyR* mutant. Mid-exponential phase cultures were adjusted to ∼10^8^ CFU/ml and diluted by dilution factor (10-fold serial dilution), and 5 μl of each dilution was spotted onto LB plates. Photos were taken 24 h after plating. CAT, catalase (2,000 Units/ml). Experiments were performed at least four times, and representative results were presented. **(E)** Disk diffusion assay for *tert*-butyl hydroperoxide (*t*-BHP). Paper disks loaded with 10 μl of 5 M *t*-BHP. In panels **B**, **C**, **E**, asterisks indicate statistically significant differences of the values compared (*n* = 4; ns, not significant; **p < 0.01; and ***p < 0.001).

In many bacteria, OxyR-binding motifs have been determined. Given sequence and function conservation, it is not surprising that these motifs are highly similar (Wan et al., 2018). To predict the operons under the direct control of *Yp*OxyR, we constructed a matrix for the OxyR-binding motif with verified OxyR-binding sequences from closely related bacterial species and used it to screen the entire genome of YPIII by RSAT (Medina-Rivera et al., 2015). In total, 38 putative *Yp*OxyR-binding sites were identified with weight values greater than 7, an arbitrary cutoff implemented in RSAT (Supplementary Table 2). On the top of the list are well-established OxyR regulon members, including *katG*, *katE*, *ahpC*, *trxB*, *grxA*, *dps*, and *trxC*, supporting that *Yp*OxyR recognizes a similar DNA motif (Figure 2A). For the remaining genes on the list, we supposed that many of them may not belong to the *Yp*OxyR regulon because weight values of their binding motifs are relatively low (≤10). But this requires further verification.

To determine the impacts of OxyR on oxidative stress response, an *oxyR* in-frame deletion strain (Δ*oxyR*) was constructed and characterized in YPIII. In liquid LB, Δ*oxyR* grew indistinguishably from the wild type (Supplementary Figure 5), indicating that *Yp*OxyR is not required for normal growth. We then assessed the role of *Yp*OxyR in combating oxidative stress. Disk diffusion assays demonstrated that Δ*oxyR* was more sensitive to H_2_O_2_, generating inhibition zones that were substantially larger than those of the wild type (Figure 2B). The phenotype can be confidently attributed to the loss of OxyR given successful genetic complementation. To provide further support to this, we compared the mutant and the wild type with respect to H_2_O_2_ consumption. Cells at the mid-exponential phase were collected, adjusted to identical densities, and incubated with 0.2 mM H_2_O_2_. As shown in Figure 2C, the Δ*oxyR* strain degraded H_2_O_2_ at a rate much lower than the wild type did; 14% and 49% of H_2_O_2_ remained 2 min after the reaction started for the wild type and the mutant, respectively. These results indicate that the *oxyR* deletion greatly impaired the ability of YPIII to remove exogenous H_2_O_2_. Furthermore, we also confirmed that the Δ*oxyR* strain carried a plating defect on LB agar plates (Figure 2D), a common phenotype of the *oxyR* mutants in bacteria such as *E. coli* and *S. oneidensis* because of compromised H_2_O_2_-scavenging capacity (Jiang et al., 2014). As exogenous catalase completely suppressed the plate defect of the Δ*oxyR* strain, it is reasonable to propose that the same mechanism is responsible for the YPIII *oxyR* mutant (Jiang et al., 2014; Shi et al., 2015).

In many bacteria lacking Ohr, such as *E. coli* and *S*. Typhi, OxyR also mediates cellular response to OPs (Imlay, 2015). Clearly, this is also true in the case of YPIII because the *oxyR* mutant exhibited significantly increased sensitivity to *t-*BHP, a widely used representative OP (Figure 2E). All together, these data conclude that YPIII employs transcriptional regulator OxyR to mediate an oxidative stress response.

### KatE Is the Primary Catalase in YPIII Positively Regulated by OxyR

**FIGURE 3:**
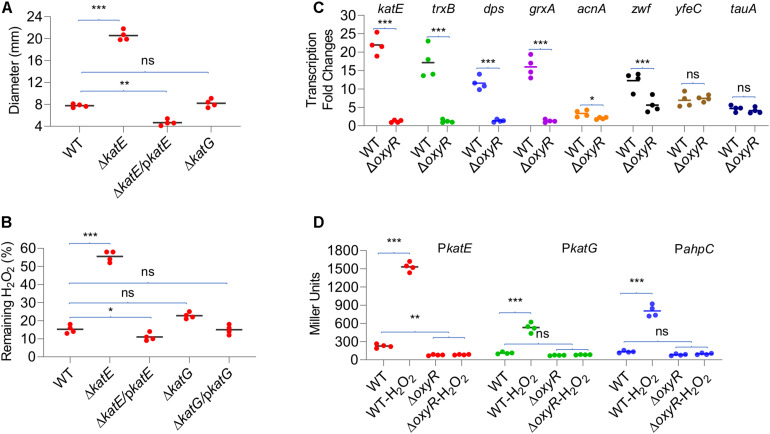
Role of catalases in decomposition of H_2_O_2_. **(A)** Disk diffusion assay of catalase mutants. **(B)** Measuring H_2_O_2_-scavenging rate of catalase mutants. **(C)** qRT-PCR analysis of transcription differences upon *oxyR* deletion. Genes with high-confident (*katE*, *trxB*, *dps*, and *grxA*) and low-confident (*acnA* and *zwf*) OxyR-binding motifs and without (*yfeC* and *tauA*) were examined. Signal intensities were processed, and the fold changes [values of the H_2_O_2_-treated wild type (WT) and Δ*oxyR*/values of the untreated WT] were calculated according to the method described in *Materials and Methods*. **(D)** Impacts of OxyR on the expression of *katE*, *katG*, and *ahpC*. Cells of mid-exponential phase before and 20 min after the H_2_O_2_ treatment were harvested for measuring the β-galactosidase assays using an integrative *lacZ* reporter. The activity of β-galactosidase represents the activity of indicated promoters. In all panels, asterisks indicate statistically significant differences of the values compared (*n* = 4; ns, not significant; *p < 0.05; **p < 0.01; and ***p < 0.001).

In general, multiple catalases are encoded in a bacterial genome, but only one exhibits predominant H_2_O_2_-scavenging activity *in vivo* (Mishra and Imlay, 2012). To test whether this also holds true in YPIII, we assessed the contribution of catalases to decomposition of H_2_O_2_. Strains lacking *katE* and *katG* were generated and characterized with the disk diffusion assay and the H_2_O_2_-decomposing assay. As shown in Figure 3A, the *katE* mutant (Δ*katE*) was hypersensitive to H_2_O_2_, generating inhibition zones that were approximately two and a half times larger than those of the wild type. On the contrary, the difference in zone sizes between the wild type and Δ*katG* was insignificant. Consistent with the results in disk diffusion assay, the *katE* deletion resulted in severely impaired ability to decompose H_2_O_2_, with nearly 60% of H_2_O_2_ remaining 2 min after the reaction started (Figure 3B). In the case of Δ*katG*, the assay revealed that this mutation slightly but still significantly compromised the ability of YPIII to decompose H_2_O_2_ when compared with that of Δ*katE*, validating that KatG is also functioning in YPIII.

Impacts of *Yp*OxyR on the expression of some of its regulon members predicted by the *in silico* analysis, including four high-confident (weight value > 12; *trxB*, *dps*, *katE*, and *grxA*) and two low-confident genes (weight value ≈9; *acnA* and *zwf*; Table 2 and Supplementary Table 2), were assessed by qRT-PCR. In cells of Δ*oxyR* prepared the same as for transcriptomics analysis, we found that none of the four high-confident genes were responsive to the H_2_O_2_ treatment, whereas two control genes, *yfeC* and *tauA*, which lack a predicted OxyR-binding motif, were upregulated upon exposure to H_2_O_2_ as in the wild type (Figure 3C). In the case of *acnA* and *zwf*, however, we found that the loss of OxyR negatively affected but not completely abolished their transcription in response to H_2_O_2_ (Figure 3C). Apparently, these data support that the predicted DNA motifs largely determine OxyR-dependent transcription.

Given the central role of catalases in protecting cells from H_2_O_2_ damage, we further compared the expression of *katE* and *katG* genes in the wild type and Δ*oxyR* strains by measuring activities of *katE* and *katG* promoters with an integrative *lacZ* reporter system (Figure 3D). In line with the transcriptomics data, the expression of *katE* and *katG* increased nearly six-fold in the wild-type cells when challenged by H_2_O_2_. While in the Δ*oxyR* strain, both genes were no longer responsive to H_2_O_2_, being expressed at levels even lower than those observed in the untreated wild-type cells. In summary, these data validate that expression of both KatE and KatG is under the positive control of OxyR, and KatE functions as the primary catalase to cope with H_2_O_2_ stress.

### AhpC Possesses Scavenging Activity Against H_2_O_2_ but Not Organic Peroxides

**FIGURE 4:**
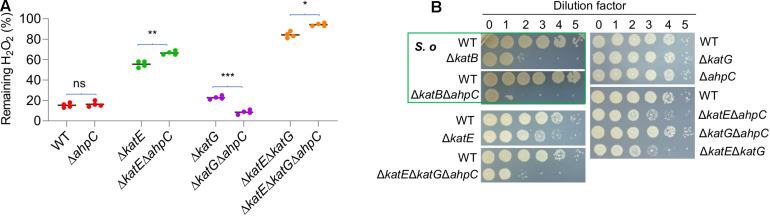
Role of AhpC in decomposition of H_2_O_2_. **(A)** Measuring the scavenging rate of H_2_O_2_ in indicated strains. Asterisks indicate statistically significant differences of the values compared (*n* = 4; ns, not significant; *p < 0.05; **p < 0.01; and ***p < 0.001). **(B)** Plating defects of the indicated strains. *S. oneidensis* represents *S. oneidensis*, which is shown for comparison. Strains outside the green box are YPIII. Experiments were performed at least four times, and representative results were presented.

The genomics analysis as presented above indicates that YPIII possesses an atypical AhpR, whose AhpF is missing. Like *katE* and *katG*, the *ahpC* gene is positively regulated by OxyR (Figure 3C), and therefore, the protein is likely produced when OxyR is activated. Given the importance of AhpR in scavenging both H_2_O_2_ and OPs, we reasoned that AhpC may be still functional in YPIII because it can also be reduced by TrxB and/or glutathione (GSH) reductase (Gor), albeit low in efficiency (Feng et al., 2020). To test this, we knocked out the *ahpC* gene from mutants lacking KatE, KatG, or both. The removal of AhpC alone had no detectable impact on H_2_O_2_ consumption (Figure 4A), an expected result because of the dominance of catalase in H_2_O_2_ degradation (Mishra and Imlay, 2012). In the absence of KatE, the effect of the AhpC loss became significant, indicating that AhpC does have some capacity of H_2_O_2_ degradation. Compared to Δ*katE*Δ*ahpC*, the loss of both catalases (Δ*katE*Δ*katG*) significantly impaired the ability of YPIII to decompose H_2_O_2_ (Figure 4A). Furthermore, additional removal of AhpC from Δ*katE*Δ*katG* nearly completely abolished the H_2_O_2_-decomposing capacity of YPIII. Therefore, these data conclude that all of KatE, KatG, and AhpC are capable of decomposing H_2_O_2_ in YPIII. Intriguingly, we found that the strain lacking both KatG and AhpC had stronger H_2_O_2_-decomposing capacity than the wild type. All of these data were verified by genetic complementation, in which one of the missing genes was expressed *in trans* in deletion mutants (Supplementary Figure 6).

To provide additional evidence, we then examined if the removal of catalases and/or AhpC would cause YPIII a plating defect, as this is also the case with *S. oneidensis* (Shi et al., 2015). Similar to an *S. oneidensis katB* (encoding dictating catalase) mutant, the Δ*katE* strain of YPIII exhibited a plating defect, albeit much less severe (Figure 4B). In contrast, the removal of either *katG* or *ahpC* did not introduce a significant defect, supporting that KatE alone is sufficient to protect cells from the killing of H_2_O_2_ generated spontaneously on the plates (Shi et al., 2015). Compared to the Δ*katE* strain, the additional removal of either *katG* or *ahpC* only marginally deteriorated the defect (Figure 4B). However, the triple mutant (Δ*katE*Δ*katG*Δ*ahpC*) exhibited significantly further lowered viability, resembling the scenario observed with the *S. oneidensis* Δ*katB*Δ*ahpC* strain. Given that *S. oneidensis* AhpC alone is responsible for cleaning endogenous H_2_O_2_, these data suggest that YPIII would require both KatG and AhpC to be a functional equivalence.

AhpC has been identified as a primary OP scavenger in many bacteria lacking Ohr, such as *E. coli*, *B. subtilis*, and *S*. Typhi (Niimura et al., 1995; Antelmann et al., 1996). We therefore predicted that AhpC is likely involved in combating against OPs in YPIII. To test this, we compared the abilities of the wild type and strains lacking catalase and/or AhpC to scavenge *t-*BHP. Results revealed that the removal of any of the genes under test (*katE*, *katG*, and *ahpC*) or all together did not introduce a significant difference in YPIII susceptibility to *t-*BHP (Supplementary Figure 7A). Quantification of the remaining *t*-BHP in the reaction also showed that the *t-*BHP-scavenging capacity of YPIII was not affected by any of these mutations or combined (Supplementary Figure 7B). In summary, these data suggest that AhpC in YPIII is able to decompose H_2_O_2_ but not OPs.

### Loss of AhpC and KatG Together Activates OxyR

**FIGURE 5:**
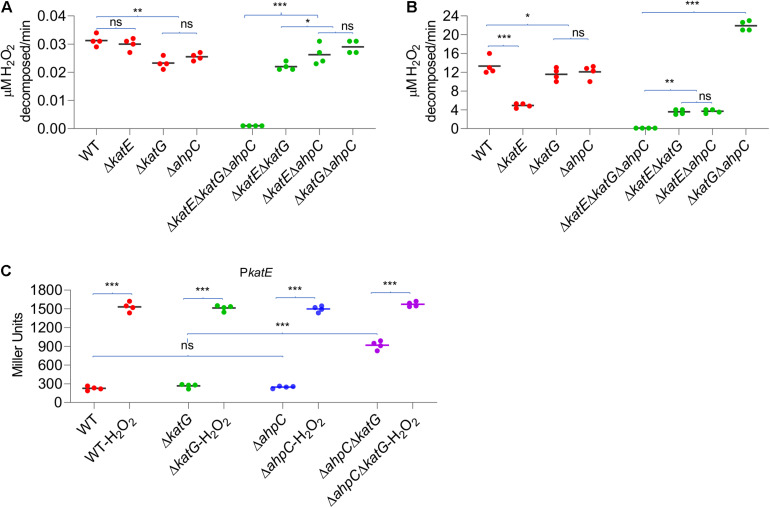
Simultaneous loss of AhpC and KatG increases H_2_O_2_ resistance. Efficiencies of AhpC, KatE, and KatG at different H_2_O_2_ concentrations. H_2_O_2_ was added at a final concentration of 2 μM **(A)** and 150 μM **(B)** to cultures of YPIII strains indicated. 2 min after addition of H_2_O_2_, the H_2_O_2_ concentration was measured. **(C)** KatE expression in strains indicated before and after the H_2_O_2_ treatment. In all panels, asterisks indicate statistically significant differences of the values compared (*n* = 4; ns, not significant; *p < 0.05; **p < 0.01; and ***p < 0.001).

The data presented thus far have concluded that in YPIII, KatE is the primary catalase in combating H_2_O_2_-induced oxidative stress, whereas both KatG and AhpC contribute. It has been established that catalase functions to scavenge high concentrations of H_2_O_2_, whereas AhpC is a more kinetically efficient scavenger of trace H_2_O_2_ (Seaver and Imlay, 2001). To determine whether the YPIII H_2_O_2_-scavenging enzymes act in a similar manner, we measured the rates at which cells decomposed low (2 μM) and high (150 μM) concentrations of H_2_O_2_. The result showed that the *katE* mutant scavenged 2 μM H_2_O_2_ significantly more rapidly than Δ*katG* and Δ*ahpC* strains did (Figure 5A), indicating that in wild type, both of KatG and AhpC carry out the decomposition of low-dose H_2_O_2_ mostly. Interestingly, a Δ*katG*Δ*ahpC* strain exhibited a faster rate in reducing 2 μM H_2_O_2_ than strains without either of these two proteins. Given that the additional removal of KatE totally disabled the Δ*katG*Δ*ahpC* strain, this observation implies that the loss of KatG and AhpC induces the expression of KatE. On the contrary, with 150 μM H_2_O_2_, the *katE* mutant worked very poorly (Figure 5B), an expected result from a strain without the primary catalase. While both Δ*katG* and Δ*ahpC* strains showed normal scavenging activity, the Δ*katG*Δ*ahpC* strain decomposed 150 μM H_2_O_2_ nearly two times faster than did the wild type, supporting an increased production of KatE.

In *E. coli*, the removal of AhpC results in H_2_O_2_ accumulation, leading to activation of OxyR and thereby increased catalase production (Seaver and Imlay, 2001). Although this is apparently not the case in YPIII, the data presented above imply that the loss of both KatG and AhpC is likely able to stimulate the production of KatE. To test this, we assessed the effects of depletion of KatG, AhpC, and both on KatE expression. As shown in Figure 5C, the *katE* gene was expressed indistinguishably in the wild-type, Δ*ahpC*, and Δ*katG* strains, and the same scenario was observed in H_2_O_2_-treated cells. These observations indicated that the loss of either AhpC or KatG alone does not significantly influence KatE production, eliminating the possibility that neither its absence is sufficient to activate OxyR in YPIII. Conversely, in the Δ*katG*Δ*ahpC* cells grown under normal aerobic conditions, we found that the *katE* promoter activity increased 3.7 times (Figure 5C). Despite this, activity of the *katE* promoter can be elevated further under the treatment of H_2_O_2_ (Figure 5C), implying that the oxidative stress risen from the loss of KatG and AhpC probably activates only a portion of OxyR molecules, which is consistent with our earlier proposal that the ratio between oxidized and reduced OxyR is in a dynamic equilibrium (Wan et al., 2018, Wan et al., 2019).

## Discussion

Resistance to oxidative stress resulting from ubiquitous ROS generated both endogenously and exogenously belongs to key virulent factors of bacterial pathogens. To deal with H_2_O_2_-induced oxidative stress, Gram-negative bacteria widely employ OxyR to sense the oxidant and mediate transcription of their regulon in a concerted manner (Imlay, 2013). Although regarded as a pleiotropic regulator, OxyR has evolved to consistently possess operons that are involved in H_2_O_2_ decomposition and damage control as the core member of regulons (Imlay, 2015). This is seemingly the case in *Y. pseudotuberculosis* YPIII. Our result in Supplementary Table 2 predicted that all H_2_O_2_-scavenging enzymes, iron-sequestering proteins, and thioredoxin and glutathione antioxidant systems are encoded by operons whose promoter regions contain most conserved OxyR-binding motifs with the highest similarities. OxyR of YPIII, highly similar to its *E. coli* counterpart in terms of sequence identity (88%) and the almost identical binding motifs, exhibits stimulating activity only (Zheng et al., 1998). This gains support from our transcriptomics data, which reveal elevated transcription of all predicted OxyR regulon members in the H_2_O_2_-treated cells.

In addition to OxyR regulon members, genes for sulfur species transport and metabolism and iron–manganese transport system are among the top up-regulated in the H_2_O_2_-treated cells. Sulfur species, especially hydrogen sulfide (H_2_S) and cysteine, are well recognized as important factors in bacterial oxidative stress response. These reductants have been shown to play a critical role in the detoxification of H_2_O_2_ in the periplasm (Ohtsu et al., 2010; Ezraty et al., 2017). Intracellular sulfur homeostasis should be carefully maintained because H_2_S and cysteine in vast excess promote oxidative damages by inhibiting catalases (Park and Imlay, 2003; Wu et al., 2015). Consistently, all components of thiol-based antioxidant systems of YPIII are most highly induced upon exposure to H_2_O_2_, conceivably requiring an active metabolism and fast shuttling of sulfur species (Feng et al., 2019). In parallel, genes for iron/manganese transport system are highly induced by exogenous H_2_O_2_. In bacteria, many enzymes using iron as a cofactor may become inactive due to the loss of the metal upon oxidative stress (Anjem et al., 2009; Anjem and Imlay, 2012). A common way to overcome this is to replace iron with manganese, and hence more manganese is imported when cells are challenged by H_2_O_2_ (Imlay et al., 2019). Although further investigations are needed, all of these observations suggest that sulfur species and manganese are important for YPIII to combat oxidative stress.

Like *E. coli*, YPIII contains two catalases: HPII KatE and HPI KatG (Mishra and Imlay, 2012). Although both KatE and KatG are highly induced upon H_2_O_2_ treatment, we first anticipate that YPIII uses KatG as the predominant H_2_O_2_-scavenging enzyme because *E. coli* does so (Seaver and Imlay, 2001). Unexpectedly, it is KatE that confers the H_2_O_2_-decomposing ability at high concentrations in YPIII. Instead, KatG exhibits a much minor but still significant contribution in scavenging high-dose H_2_O_2_. More importantly, KatG shows a strong scavenging activity toward low concentrations of H_2_O_2_, a role that is exclusively played by AhpC in *E. coli*. Despite this, given that *Yp*OxyR becomes activated in the absence of both AhpC and KatG, but not of either one, it is clear that both KatG and AhpC, while neither is sufficient, is able to scavenge low concentrations of H_2_O_2_ in YPIII. It is well established that AhpC allows *E. coli* cells to degrade low concentrations of H_2_O_2_ because of its high kinetic efficiency (*k*_cat_/*K*_M_, ∼10^–8^ M^–1^ s^–1^; Mishra and Imlay, 2012). We believe that this is also likely true in the case of YPIII KatG. But the result of impaired H_2_O_2_-scavenging ability of Δ*katG* and Δ*ahpC* at low levels in YPIII implies that YPIII KatG possesses a kinetic efficiency similar to, or at least not significantly lower than, that of AhpC, the catalytic activity that is substantially greater than those of KatE and KatG in *E. coli* whose activities are similar to each other (∼10^–6^ M^–1^ s^–1^; Obinger et al., 1997; Hillar et al., 2000). We are working to test this notion.

Bacterial AhpC is regarded as the founding member of the peroxiredoxin family, and its homologs appear to be more widely distributed than catalase/peroxidase (Chae et al., 1994). The AhpR complex is highly conserved among diverse bacteria, with respect to not only amino acid sequence but also gene organization (Feng et al., 2020). To date, all bacteria that employ OxyR as the master H_2_O_2_-responding regulator are equipped with an *ahp* operon encoding both AhpC and its cognate reductase AhpF, including *M. tuberculosis* in which AhpF is replaced by AhpD to reduce AhpC (Baker et al., 2001; Jaeger et al., 2004). Apart from that, AhpC without a co-transcribed cognate reductase is found in *H. pylori*, a gastric pathogen that represents an extreme example in terms of oxidative stress response machinery because it lacks homologs of the oxidative stress regulators present in other bacteria, including OxyR, SoxR, SoxS, and PerR (Baker et al., 2001; Wang et al., 2006). A sequence alignment reveals that AhpC of *Y. pseudotuberculosis* is more closely related to typical AhpCs than to the *H. pylori* AhpC: there are significantly more identical residues in AhpCs of *E. coli*, *S. oneidensis*, and *Y. pseudotuberculosis* (Supplementary Figure 8). Despite this, we also observed that the linear arrangement of AhpC gene is highly conserved in *Yersinia* species (Supplementary Figure 9), a phenomenon that is quite rare in other bacteria. Therefore, it would be interesting to demystify how evolution has honed this particular phenomenon.

**FIGURE 6:**
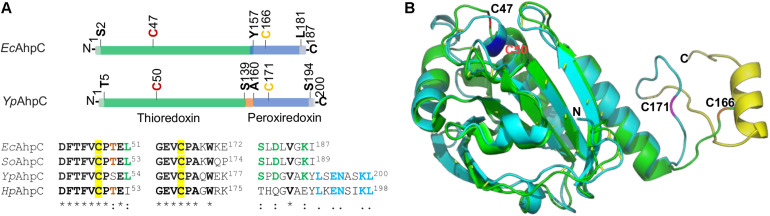
Comparative analysis of AhpC proteins. **(A)** The domain structure of *Ec*AhpC and *Yp*AhpC. Both proteins are composed of two domains, thioredoxin (green) and peroxiredoxin (blue), with peroxidatic Cys47 (or Cys50) and resolving Cys166 (or Cys171) in red and yellow, respectively. Sequence alignment of four representative AhpCs shown below demonstrates the conservation of regions covering two active Cys residues (identical residues are in bold with star mark) and the *C*-terminal tail region. Conserved residues in three out of four AhpCs are shown in red and green; the identical residues in *Yp*AhpC and *Hp*AhpC are shown in blue. **(B)** Structural comparison of *Yp*AhpC and *Ec*AhpC (PDB accession number 4o5r). Shown is a superimposition of *Yp*AhpC (cyan) and *Ec*AhpC (green). The α helix formed by the *C*-terminal tail of *Yp*AhpC is shown in yellow. Active Cys residues are labeled.

Although the reductase for YPIII AhpC remains to be determined, we may get clues from *H. pylori* AhpC in terms of their differences in sequence and structure from the typical ones (with AhpF as the cognate reductase). Reduction of *H. pylori* AhpC is carried out by thioredoxin TrxA, which is reduced by thioredoxin reductase TrxR (also called TrxB) using NADPH as the electron donor (Wang et al., 2006). A common feature that *Yp*AhpC and *Hp*AhpC share is that they have an additional eight-residue segment in the *C*-terminal end, which is missing in the typical AhpC proteins (Figure 6A and Supplementary Figure 8). In fact, the last few residues in the *C* terminus of AhpC have been shown to be particularly critical in maintaining an enzymatically active AhpR complex *in vitro* (Dip et al., 2014a). This *C*-terminal tail of AhpC is crucial in complexation of AhpC with AhpF, TrxR, or glutathione-disulfide reductase GorA (Dip et al., 2014a; Feng et al., 2020). Coincidently, earlier studies of certain *E. coli* and *S. oneidensis* AhpC mutants have established that certain mutations, especially in the *C*-terminal region, can transform AhpC to a surprisingly malleable enzyme, reducible not only by Trx system but also by Grx system (Faulkner et al., 2008; Feng et al., 2020). Therefore, we speculate that the extra eight residues at the *C*-terminal end of YPIII AhpC may affect the specificity of the protein for its reducing partner.

The difference in the *C*-terminal region may also provide an explanation for the finding that YPIII AhpC does not have the ability to reduce OPs. It has been shown that catalytic efficiency of AhpC with small hydroperoxides, especially H_2_O_2_, is substantially higher (∼100-fold) than that with bulky, tertiary hydroperoxides, such as *t*-BHP (Parsonage et al., 2008). For both types of oxidants, the catalytic reaction adopts a binding and releasing mechanism that enables the assembly of AhpC and the reducing partner to undergo efficient catalytic cycles of transferring electrons without compromising the catalytic turnover rate (Parsonage et al., 2015). A prerequisite for such a mechanism is the correct fitting of the *C*-terminal tail of AhpC into the “arch-like” groove on the binding surface of the reducing partner, resulting in formation of an active-site pocket, to which small peroxides are more accessible (Dip et al., 2014b). The additional *C*-terminal tail of YPIII AhpC may completely prevent large OPs from entering the active-site pocket (Figure 6B). Compared to the *E. coli* AhpC structure (PDB accession number 4o5r; Dip et al., 2014b), *Y. pseudotuberculosis* AhpC has an α-helix that is formed by the extra residues. Unfortunately, how this α-helix folds remains unknown because the available *H. pylori* AhpC structure is truncated without this tail (PDB accession number 1zof; Papinutto et al., 2005; Supplementary Figure 10). But one may imagine that the extra *C*-terminus α-helix may hinder the reduction of OPs. Given that *Yp*AhpC, unlike typical AhpCs, is not sufficient to scavenge endogenous H_2_O_2_, we speculate that the α-helix also compromises the efficiency of H_2_O_2_ reduction. If this holds, the difference in the kinetic efficiencies for H_2_O_2_ between AhpC and catalases of YPIII is likely significantly smaller than that between the *E. coli* counterparts.

## Data Availability Statement

The datasets presented in this study can be found in online repositories. The names of the repository/repositories and accession number(s) can be found in the article/Supplementary Material.

## Author Contributions

FW and HG designed and supported the research. FW, XF, and JY performed the research and analyzed the data. FW, XF, and HG wrote the manuscript. All authors contributed to the article and approved the submitted version.

## Conflict of Interest

The authors declare that the research was conducted in the absence of any commercial or financial relationships that could be construed as a potential conflict of interest.

## Footnotes

1 http://www.sbg.bio.ic.ac.uk/∼phyre/
