# Supplementary material for: Distinct H2O2-Scavenging System in Yersinia pseudotuberculosis: KatG and AhpC Act Together to Scavenge Endogenous Hydrogen Peroxide
Source: Front Microbiol. 2021 May 7;12:626874. doi: 10.3389/fmicb.2021.626874 (PMC8139631; doi:10.3389/fmicb.2021.626874)
Supplement: Supplementary file 1 [file Data_Sheet_1.pdf]

```
EcOxyR     MNIRDLEYLVALAEHRHFRRAADSCHVSQPTLSGQIRKLEDELGVMLLERTSRKVLFTQA  60
YpOxyR     MNIRDLEYLVALAEFRHFRRAADSCHVSQPTLSGQIRKLEDELGIMLLERTSRKVLFTQA  60
           **************.*****************************:***************

EcOxyR     GMLLVDQARTVLREVKVLKEMASQQGETMSGPLHIGLIPTVGPYLLPHIIPMLHQTFPKL  120
YpOxyR     GLLLVEQAKTVLREVKVLKEMASLQGESMSGPLHIGLIPTVGPYLLPQIIPMLHKTFPKL  120
           *:***:**:************** ***:*******************:******:*****

EcOxyR     EMYLHEAQTHQLLAQLDSGKLDCVILALVKESEAFIEVPLFDEPMLLAIYEDHPWANREC  180
YpOxyR     EMYLHEAQTQNLLAQLDSGKLDCAILALVKETEAFIEIPLFDEPMNLAIYADHPWANRER  180
           *********::************.*******:*****:******* **** ********

EcOxyR     VPMADLAGEKLLMLEDGHCLRDQAMGFCFEAGADEDTHFRATSLETLRNMVAAGSGITLL  240
YpOxyR     VEMHELAGEKLLMLEDGHCLRDQAMGFCFQAGADEDTHFRATSLETLRNMVAAGSGITLL  240
           * * :************************:******************************

EcOxyR     PALAVPPERKRDGVVYLPCIKPEPRRTIGLVYRPGSPLRSRYEQLAEAIRARMDGHFDKV  300
YpOxyR     PALAVPNERQRDGVCYLECYKPVPKRTIALVYRPGSPLRGRYEQLAEAIRDHMQERMAPS  300
           ****** **:**** ** * ** *:***.**********.********** :*:  ::

EcOxyR     LKQAV      305
YpOxyR     LEQAI      305
           *:**:
```

**Fig. S1.** Sequence alignment of OxyR proteins. Oxidative residues are highlighted. *Yp*, *Y. pseudotuberculosis*; *So*, *S. oneidensis*; *Ec*, *E. coli*.

```
SoOxyR      MKNLPSLKNLYYLVNLHQEQNFNRAAKVCFVSQSTLSSGIQNLEEQLGHQLIERDHKSFM  60
EcOxyR      ----MNIRDLEYLVALAEHRHFRRAADSCHVSQPTLSGQIRKLEDELGVMLLERTSRKVL  56
YpOxyR      ----MNIRDLEYLVALAEFRHFRRAADSCHVSQPTLSGQIRKLEDELGIMLLERTSRKVL  56
PaOxyR      ----MTLTELRYIVTLAQEQHFGRAAERCHVSQPTLSVGVKKLEDELGVLIFERSKSAVR  56
NmOxyR      ----MTLTELRYIVAVAQERHFGRAARRCFVSQPTLSIAIKKLEEELAVSLFDRSSNDII  56
                 .: :* *:* : : ::* ***  *.*** ***  :::**::*.  :::*    .

SoOxyR      FTAIGEEVVQRSRKILTDVDDLVELVKNQGEPMTGDIRLGCIPTIAPFLLSRVVKQCQQA  120
EcOxyR      FTQAGMLLVDQARTVLREVKVLKEMASQQGETMSGPLHIGLIPTVGPYLLPHIIPMLHQT  116
YpOxyR      FTQAGLLLVEQAKTVLREVKVLKEMASLQGESMSGPLHIGLIPTVGPYLLPQIIPMLHKT  116
PaOxyR      LTPVGEGIVAQAQKVLEQAQGIRELAQAGKNQLAAPLKVGAIYTIGPYLFPHLIPQLHRV  116
NmOxyR      TTEAGERIVAQARKVLEEAELIRHLANEEQNELEGAFKLGLIFTVAPYLLPKLIVSLRRT  116
             *  *  :* :::.:* :.. : .:..   : : . :::* * *:.*:*: :::   ::.

SoOxyR      YPEMSLLLKEDTTERLLDALGKGELDLLILALPVDTSGYHSMKVGIDPFKMVIHKDLVGG  180
EcOxyR      FPKLEMYLHEAQTHQLLAQLDSGKLDCVILALVKESEAFIEVPLFDEPMLLAIYEDHPWA  176
YpOxyR      FPKLEMYLHEAQTQNLLAQLDSGKLDCAILALVKETEAFIEIPLFDEPMNLAIYADHPWA  176
PaOxyR      APQMPLYIEENFTHILRDKLRTGELDAIIIALPFQEADVLTKPLFDEPFYVLMPADHPWT  176
NmOxyR      APKMPLMLEENYTHTLTESLKRGDVDAIIVAEPFQEPGIVTEPLYDEPFFVIVPKGHSFE  176
             *:: : :.*  *. *   *  *.:*  *:*   :         :  :*: : :  .

SoOxyR      IHQPIDYQTLPDESIFLLQSEHCITGHAITACQLGDSAK-----VNPFAATSLHTLVQMV  235
EcOxyR      NRECVPMADLAGEKLLMLEDGHCLRDQAMGFCFEAGADED-----THFRATSLETLRNMV  231
YpOxyR      NRERVEMHELAGEKLLMLEDGHCLRDQAMGFCFQAGADED-----THFRATSLETLRNMV  231
PaOxyR      AKASIDSELLNDKSLLLLGEGHCFRDQVLEACPTVRKGDE--NKHTTVESSSLETIRHMV  234
NmOxyR      ELDAVSPRMLGEEQVLLLTEGNCMRDQVLSSCSELAAKQRIQGLTNTLQGSSINTIRHMV  236
                :    *  :.:::* . :*: .:.:  *      .      . . .:*:.*: :**

SoOxyR      NSKLGTTFLPQMAIDAGILNDTDLVVMTPPGEAPYRDIGLVWRQTTSRILTFRTLGLLIQ  295
EcOxyR      AAGSGITLLPALAVPPERKRDGVV-YLPCIKPEPRRTIGLVYRPGSPLRSRYEQLAEAIR  290
YpOxyR      AAGSGITLLPALAVPNERQRDGVC-YLECYKPVPKRTIALVYRPGSPLRGRYEQLAEAIR  290
PaOxyR      ASGLGVSVLPFSAVDSHHYAPGVIEVRPFSAPVPFRTVAIAWRASFPRPRAIEVLADSIR  294
NmOxyR      ASGLAISVLPATALTEND--HMLFSIIPFEGTPPSRRVVLAYRRNFVRPKALSAMKAAIM  294
             :  . :.**  *:                   * * : :.:*           :   *

SoOxyR      KLLTNETAQ-------     304
EcOxyR      ARMDGHFDKVLKQAV-     305
YpOxyR      DHMQERMAPSLEQAI-     305
PaOxyR      LCSVARPQTQEQPQIA     310
NmOxyR      QSQLHGVSFIRD----     306
```

**Fig. S2.** Sequence alignment of OxyR proteins. Oxidative residues are highlighted. *Yp*, *Y. pseudotuberculosis*; *So*, *S. oneidensis*; *Ec*, *E. coli*; *Pa*, *Pseudomonas aeruginosa*; *Nm*, *Neisseria meningitidis*.

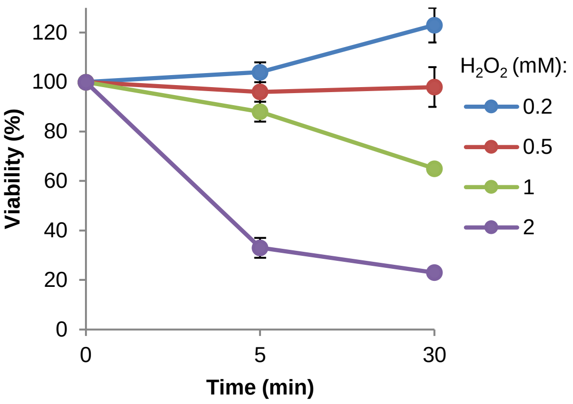


**Fig. S3.** Survival of YPIII after treated with $H_2O_2$. $H_2O_2$ was added to mid-exponentail cultures to the final concentrations as indicated. After 5 min and 30 min, samples were properly diluted and plated on LB plates. Colony counting was done after 24 h. Experiments were performed at least four times, and the data reported represent the mean ($n$ = 4) ± standard deviation (SD).

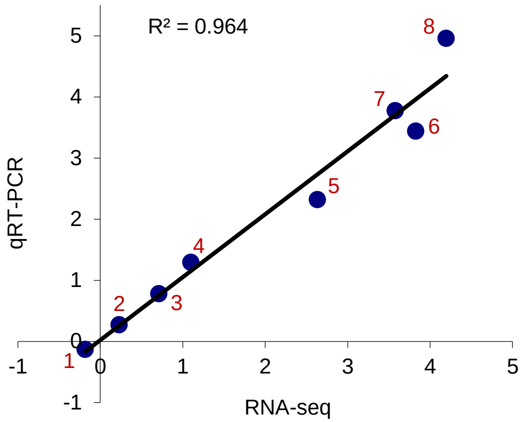


**Fig. S4.** Comparison of expression measurements by RNA-seq and Real Time qRT-PCR assays. Fold changes in Log2 value of 8 genes between the $H_2O_2$-treated and control samples are shown. Genes shown are *yfeC* (1), *tauA* (2), *zwf* (3), *acnA* (4), *grxA* (5), *dps* (6), *trxB* (7), and *katE* (8).

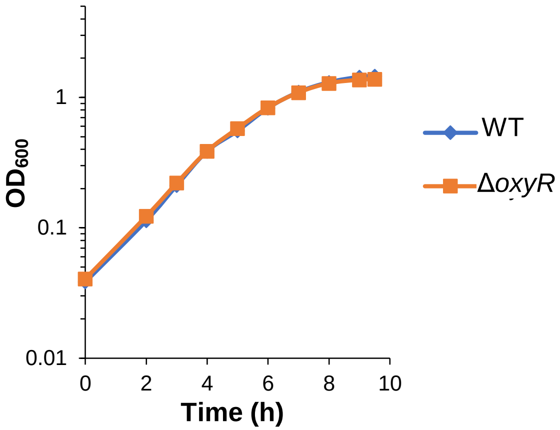


**Fig. S5.** Growth of YPIII wild-type and Δ*oxyR* in LB.

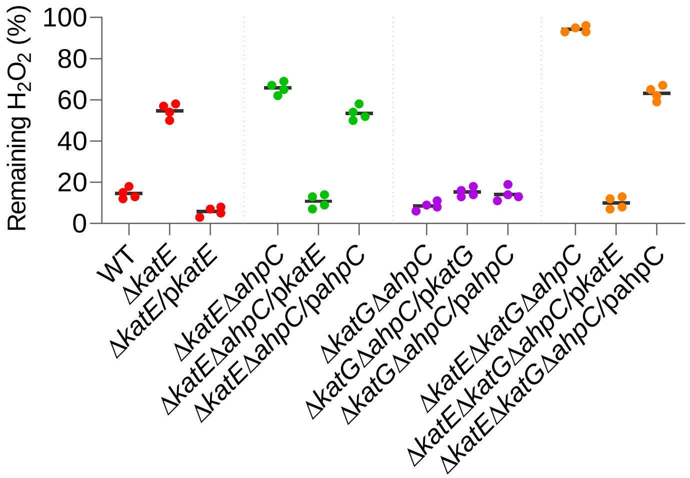


**Fig. S6.** Genetic complementation of mutants lacking catalase, AhpC, or both. A copy of the gene indicated was expressed *in trans* for complementation.

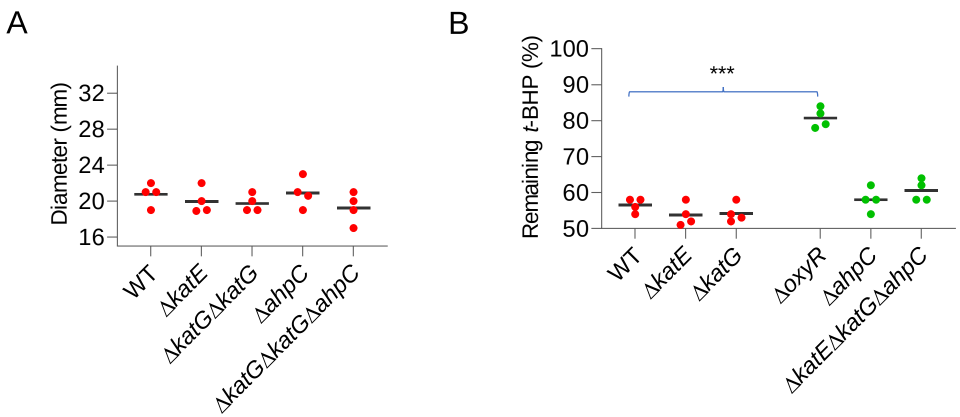


**Fig. S7.** Role of catalase and AhpC in decomposition of *t*-BHP. (A) Disk diffusion assay of indicated mutants. (B) Measuring *t*-BHP scavenging rate of indicated mutants. Asterisks indicate statistically significant difference of the values compared ($n$ = 4; *, $p < 0.05$; **, $p < 0.01$; ***, $p < 0.001$).

```
SoAhpC      MTQSIINSTIKPFKATA-YHNGEFVPV--TEQDLLGKWSVVFFYPADFTFVCPTELGDMA      57
EcAhpC      --MSLINTKIKPFKNQA-FKNGEFIEI--TEKDTEGRWSVVFFFYPADFTFVCPTELGDVA      55
YpAhpC      --MVLVTRQAPDFTAAAVLGNGEIVENFNLKKHLNGRPAVLFFWPMDFTFVCPSELIAFD      58
HpAhpC      ---MLVTKLAPDFKAPAVLGNNEVDEHFELSKNLGKNGVILFFWPKDFTFVCPTEIIAFD      57
                ::.     *.  *   *.*.      .:.  .   :.**:* *******:*:  .

SoAhpC      DHYAKLQGMGVEVYSVSTDTHFTHKAWHDTS---DTIKKINFPMLADPTGTISRNFGVMI      114
EcAhpC      DHYEELQKLGVDVYAVSTDTHFTHKAWHSSS---ETIAKIKYAMIGDPTGALTRNFDNMR      112
YpAhpC      HRYEEFQKRGVEVVGVSFDSEFVHNAWRKTPVDKGGIGEVKYPMVADIKREIQKAYGIEH      118
HpAhpC      KRVKDFHEKGFNVIGVSIDSEQVHFAWKNTPVEKGGIGQVSFPMVADITKSISRDYDVLF      117
             .: .::  *.:* .** *:. .* **:.:      * ::.: *:.* .  : : :.

SoAhpC      EEEGLALRGTFVINPEGQIKVAEIHDLGIGRSAQELVRKIQAAQYVATHDGEVCPAKWQP      174
EcAhpC      EDEGLADRATFVVDPQGIIQAIEVTAEGIGRDASDLLRKIKAAQYVASHPGEVCPAKWKE      172
YpAhpC      PDAGVALRGSFLIDKNGIVRSQIVNDLPIGRNIDEMIRTVDALQFHEE-HGEVCPAQWEK      177
HpAhpC      E-EAIALRGAFLIDKNMKVRHAVINDLPLGRNADEMLRMVDALLHFEE-HGEVCPAGWRK      175
               .:* *.:*:::  :  ::   :    :**. .:::* :.* .      ****** *.

SoAhpC      GDETLAPSLDLVGKI--------      189
EcAhpC      GEATLAPSLDLVGKI--------      187
YpAhpC      GKAGMGASPDGVAKYLSENASKL      200
HpAhpC      GDKGMKATHQGVAEYLKENSIKL      198
            *.  :  : : *.:
```

**Fig. S8.** Sequence alignment of AhpC proteins. Oxidative residues are highlighted. *So*, *S. oneidensis*; *Ec*, *E. coli*; *Yp*, *Y. pseudotuberculosis*; *Hp*, *H. pylori*. Residues in green represent identical ones in *YpAhpC*, *EcAhpC*, and *SoAhpC* while residues in red represent identical ones in *HpAhpC*, *EcAhpC*, and *SoAhpC*.

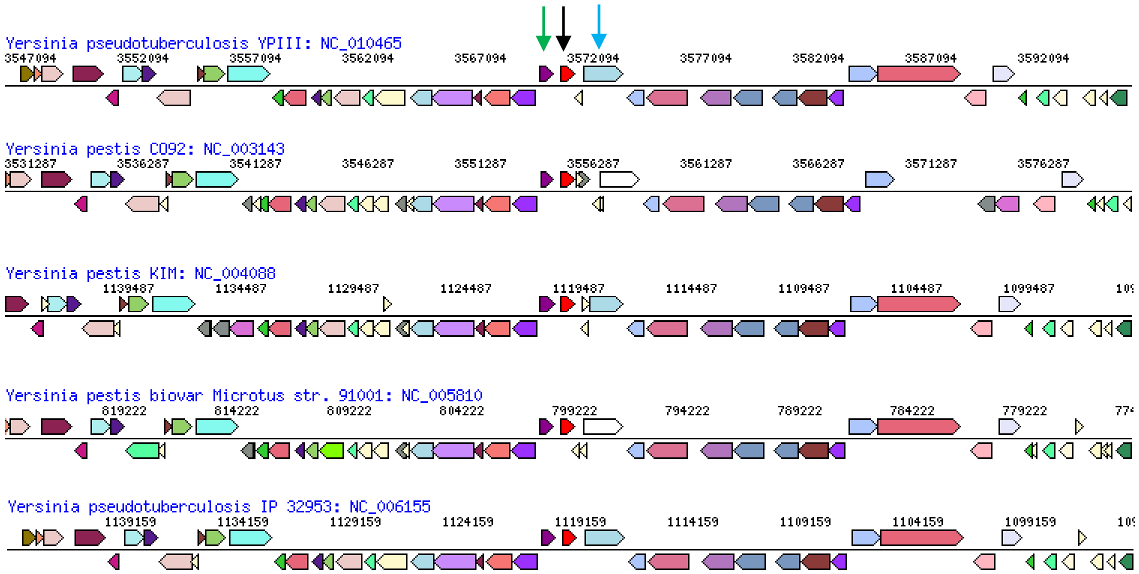


**Fig. S9.** Gene organization of the *ahpC* locus in *Yersinia* species. The *ahpC* gene is pointed by a black arrow. Genes *acpH* (green arrow, phosphodiesterase) and YPK_RS16380 (blue arrow, γ-glutamyltransferase)

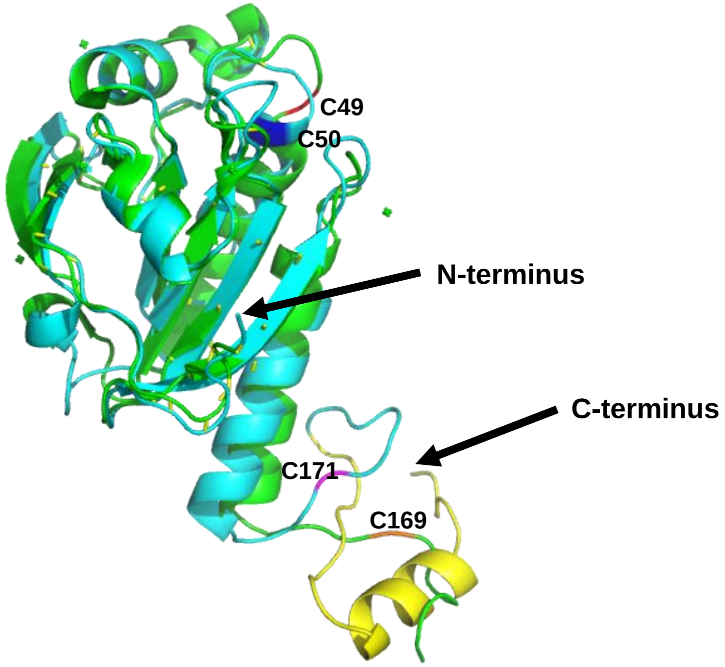


**Fig. S10.** Structural comparison of *Yp*AhpC and *Hp*AhpC (PDB accession number 1zof). Shown is a superimposition of *Yp*AhpC (cyan) and *Hp*AhpC (green). The α helix formed by the C-terminal tail of *Yp*AhpC is shown in yellow.

TABLE S1 Genes whose transcription is significantly changed in *Y. pseudotuberculosis* response to $H_2O_2$ (treated/untreated)

| Gene_id | Gene name | Gene description | FC(B/A) | Log2FC(B/ | Pvalue |
|---|---|---|---|---|---|
| YPK_RS20590 | YPK_RS20590 | glutathione peroxidase | 689.463 | 9.42933 | 0 |
| YPK_RS20595 | YPK_RS20595 | dihydrolipoyl dehydrogenase | 89.329 | 6.481053 | 0 |
| YPK_RS16760 | trxC | thioredoxin TrxC | 75.426 | 6.236988 | 0 |
| YPK_RS08095 | dps | DNA starvation/stationary phase protection protein Dps | 48.326 | 5.594717 | 0 |
| YPK_RS17250 | cysC | adenylyl-sulfate kinase | 38.451 | 5.264968 | 2.54E-83 |
| YPK_RS22535 | YPK_RS22535 | hypothetical protein | 34.038 | 5.089083 | 1.22E-28 |
| YPK_RS17260 | cysD | sulfate adenylyltransferase subunit CysD | 31.961 | 4.998246 | 1.04E-55 |
| YPK_RS17255 | cysN | sulfate adenylyltransferase subunit CysN | 28.297 | 4.822593 | 0 |
| YPK_RS14285 | YPK_RS14285 | catalase | 27.604 | 4.786793 | 0 |
| YPK_RS13675 | YPK_RS13675 | GrxA family glutaredoxin | 22.587 | 4.497435 | 0 |
| YPK_RS17265 | cobA | uroporphyrinogen-III C-methyltransferase | 17.955 | 4.166307 | 3.1E-13 |
| YPK_RS00550 | gorA | glutathione-disulfide reductase | 16.227 | 4.020291 | 0 |
| YPK_RS13735 | YPK_RS13735 | isopenicillin N synthase family oxygenase | 16.215 | 4.019288 | 7.65E-21 |
| YPK_RS13730 | YPK_RS13730 | ABC transporter substrate-binding protein | 15.426 | 3.947246 | 1.22E-24 |
| YPK_RS03760 | YPK_RS03760 | TonB-dependent receptor | 13.228 | 3.725506 | 2.52E-40 |
| YPK_RS09490 | YPK_RS09490 | CMD domain-containing protein | 13.122 | 3.71387 | 1.45E-67 |
| YPK_RS07100 | cysA | sulfate/thiosulfate ABC transporter ATP-binding protein CysA | 13.042 | 3.705091 | 2.6E-200 |
| YPK_RS16765 | YPK_RS16765 | DTW domain-containing protein | 12.712 | 3.668118 | 2E-111 |
| YPK_RS10680 | zwf | glucose-6-phosphate dehydrogenase | 12.316 | 3.622494 | 0 |
| YPK_RS07190 | cysK | cysteine synthase A | 11.887 | 3.571271 | 4.1E-43 |
| YPK_RS05645 | nrdE | class 1b ribonucleoside-diphosphate reductase subunit alpha | 11.503 | 3.523998 | 1.3E-151 |
| YPK_RS01880 | YPK_RS01880 | glucose-6-phosphate isomerase | 11.141 | 3.477837 | 0 |
| YPK_RS05655 | nrdH | redoxin NrdH | 10.935 | 3.450829 | 1.45E-32 |
| YPK_RS17035 | cybB | cytochrome b561 | 10.562 | 3.400767 | 0 |
| YPK_RS17295 | cysJ | NADPH-dependent assimilatory sulfite reductase flavoprotein subunit | 10.322 | 3.367621 | 1.29E-35 |
| YPK_RS07095 | cysW | sulfate/thiosulfate ABC transporter permease CysW | 9.93 | 3.311841 | 2.18E-39 |
| YPK_RS07085 | YPK_RS07085 | sulfate ABC transporter substrate-binding protein | 9.809 | 3.294077 | 2.96E-62 |
| YPK_RS13725 | YPK_RS13725 | methionine ABC transporter ATP-binding protein | 9.098 | 3.185484 | 7.1E-18 |
| YPK_RS05650 | nrdI | class Ib ribonucleoside-diphosphate reductase assembly flavoprotein NrdI | 9.067 | 3.180584 | 6.63E-06 |
| YPK_RS01885 | psiE | phosphate-starvation-inducible protein PsiE | 8.322 | 3.05685 | 1.7E-144 |
| YPK_RS01555 | dprA | DNA-protecting protein DprA | 8.143 | 3.025521 | 3.04E-49 |
| YPK_RS21390 | YPK_RS21390 | ABC transporter substrate-binding protein | 7.423 | 2.892095 | 2.9E-113 |

| | | | | | |
|---|---|---|---|---|---|
| YPK_RS17025 | katG | catalase/peroxidase HPI | 7.241 | 2.856275 | 0 |
| YPK_RS17030 | cybC | cytochrome b562 | 7.157 | 2.839348 | 0 |
| YPK_RS18095 | yaaA | peroxide stress protein YaaA | 7.102 | 2.828168 | 0 |
| YPK_RS00530 | YPK_RS00530 | DUF943 family protein | 7.097 | 2.827129 | 1.7E-291 |
| YPK_RS17125 | YPK_RS17125 | TonB-dependent receptor | 6.797 | 2.764826 | 4.73E-34 |
| YPK_RS10675 | eda | bifunctional 4-hydroxy-2-oxoglutarate aldolase/2-dehydro-3-deoxy-phospł | 6.605 | 2.723491 | 7.7E-231 |
| YPK_RS09105 | yfeC | iron/manganese ABC transporter permease subunit YfeC | 6.359 | 2.668913 | 9E-118 |
| YPK_RS21400 | YPK_RS21400 | amino acid ABC transporter permease | 6.112 | 2.611567 | 9.13E-25 |
| YPK_RS11135 | astE | succinylglutamate desuccinylase | 5.922 | 2.566186 | 8.7E-45 |
| YPK_RS09100 | yfeD | iron/manganese ABC transporter permease subunit YfeD | 5.84 | 2.545896 | 6.16E-82 |
| YPK_RS21395 | YPK_RS21395 | amino acid ABC transporter permease | 5.681 | 2.506159 | 1.63E-15 |
| YPK_RS20640 | argC | N-acetyl-gamma-glutamyl-phosphate reductase | 5.369 | 2.424658 | 8.3E-125 |
| YPK_RS01270 | tauC | taurine ABC transporter permease TauC | 5.279 | 2.400303 | 1.17E-05 |
| YPK_RS07090 | cysT | sulfate/thiosulfate ABC transporter permease CysT | 5.102 | 2.351124 | 3.79E-23 |
| YPK_RS01265 | tauB | taurine ABC transporter ATP-binding subunit | 4.675 | 2.224862 | 3.15E-06 |
| YPK_RS01260 | tauA | taurine ABC transporter substrate-binding protein | 4.608 | 2.204107 | 2.05E-08 |
| YPK_RS05640 | nrdF | class 1b ribonucleoside-diphosphate reductase subunit beta | 4.579 | 2.195036 | 7E-29 |
| YPK_RS20585 | oxyR | DNA-binding transcriptional regulator OxyR | 4.509 | 2.172925 | 1.5E-279 |
| YPK_RS13445 | trxB | thioredoxin-disulfide reductase | 4.461 | 2.157239 | 0 |
| YPK_RS18280 | YPK_RS18280 | DUF1435 domain-containing protein | 4.446 | 2.152613 | 0.000594 |
| YPK_RS17120 | YPK_RS17120 | ShET2/EspL2 family type III secretion system effector toxin | 4.439 | 2.150327 | 5.76E-78 |
| YPK_RS04375 | YPK_RS04375 | hemolysin III family protein | 4.331 | 2.114622 | 5.09E-13 |
| YPK_RS09110 | yfeB | iron/manganese ABC transporter ATP-binding protein YfeB | 4.277 | 2.096635 | 4.8E-101 |
| YPK_RS11005 | YPK_RS11005 | HEAT repeat domain-containing protein | 4.226 | 2.079389 | 4.7E-34 |
| YPK_RS12775 | YPK_RS12775 | hypothetical protein | 4.094 | 2.033549 | 1.83E-16 |
| YPK_RS18090 | tal | transaldolase | 4.077 | 2.027613 | 0 |
| YPK_RS13720 | metI | ABC transporter permease | 4.075 | 2.026939 | 9.64E-09 |
| YPK_RS01235 | YPK_RS01235 | DUF1240 domain-containing protein | 4.023 | 2.008218 | 0.004481 |
| YPK_RS14910 | recN | DNA repair protein RecN | 3.933 | 1.975666 | 2.5E-190 |
| YPK_RS03930 | YPK_RS03930 | lysine N(6)-hydroxylase/L-ornithine N(5)-oxygenase family protein | 3.909 | 1.966741 | 9.85E-18 |
| YPK_RS17285 | YPK_RS17285 | phosphoadenylyl-sulfate reductase | 3.788 | 1.921413 | 5.3E-183 |
| YPK_RS03935 | iucC | aerobactin synthase IucC | 3.781 | 1.918646 | 6.36E-17 |
| YPK_RS11230 | YPK_RS11230 | cytochrome c | 3.763 | 1.911757 | 0.022413 |
| YPK_RS03170 | YPK_RS03170 | hypothetical protein | 3.751 | 1.907092 | 8.37E-08 |

| | | | | | |
|---|---|---|---|---|---|
| YPK_RS11885 | tcyJ | cystine ABC transporter substrate-binding protein | 3.716 | 1.893904 | 3.3E-119 |
| YPK_RS11010 | YPK_RS11010 | hypothetical protein | 3.714 | 1.893092 | 8.15E-28 |
| YPK_RS20635 | argB | acetylglutamate kinase | 3.648 | 1.867089 | 7.35E-38 |
| YPK_RS09015 | YPK_RS09015 | L-cystine transporter | 3.615 | 1.854014 | 6.06E-80 |
| YPK_RS09115 | yfeA | iron/manganese ABC transporter substrate-binding protein YfeA | 3.605 | 1.850102 | 1.78E-73 |
| YPK_RS03055 | YPK_RS03055 | DsbA family protein | 3.578 | 1.839152 | 9.15E-11 |
| YPK_RS16370 | YPK_RS16370 | peroxiredoxin C | 3.508 | 1.81045 | 0 |
| YPK_RS19415 | lexA | repressor LexA | 3.506 | 1.809867 | 2.07E-88 |
| YPK_RS17290 | cysI | assimilatory sulfite reductase (NADPH) hemoprotein subunit | 3.429 | 1.777774 | 5E-99 |
| YPK_RS11140 | astB | N-succinylarginine dihydrolase | 3.399 | 1.765102 | 7.27E-40 |
| YPK_RS03110 | YPK_RS03110 | hypothetical protein | 3.36 | 1.748282 | 1.04E-45 |
| YPK_RS07985 | YPK_RS07985 | heme-degrading domain-containing protein | 3.289 | 1.717864 | 2.04E-05 |
| YPK_RS07670 | fabB | beta-ketoacyl-ACP synthase I | 3.246 | 1.698798 | 3.9E-163 |
| YPK_RS10200 | acnA | aconitate hydratase AcnA | 3.223 | 1.688479 | 6.8E-122 |
| YPK_RS03645 | YPK_RS03645 | transcriptional regulator | 3.212 | 1.683331 | 6.03E-15 |
| YPK_RS12530 | argG | argininosuccinate synthase | 3.195 | 1.675644 | 3E-100 |
| YPK_RS12675 | gndA | NADP-dependent phosphogluconate dehydrogenase | 3.191 | 1.674048 | 4.1E-266 |
| YPK_RS03940 | YPK_RS03940 | acetyltransferase | 3.187 | 1.672269 | 7.86E-09 |
| YPK_RS01245 | YPK_RS01245 | YheU family protein | 3.173 | 1.665822 | 7.54E-06 |
| YPK_RS12730 | YPK_RS12730 | lysine N(6)-hydroxylase/L-ornithine N(5)-oxygenase family protein | 3.166 | 1.662873 | 1.43E-30 |
| YPK_RS06045 | YPK_RS06045 | tyrosine-type recombinase/integrase | 3.148 | 1.654319 | 1.45E-76 |
| YPK_RS12725 | YPK_RS12725 | acetyltransferase | 3.136 | 1.649097 | 1.84E-10 |
| YPK_RS11185 | phoH | phosphate starvation-inducible protein PhoH | 3.118 | 1.640575 | 7.48E-31 |
| YPK_RS15570 | YPK_RS15570 | DinI family protein | 3.072 | 1.619243 | 1.03E-21 |
| YPK_RS05265 | YPK_RS05265 | amino-acid N-acetyltransferase | 3.044 | 1.606143 | 2.28E-64 |
| YPK_RS13145 | sulA | cell division inhibitor SulA | 3.004 | 1.587042 | 3.85E-49 |
| YPK_RS12735 | YPK_RS12735 | aspartate aminotransferase family protein | 2.986 | 1.578236 | 4.03E-31 |
| YPK_RS03945 | YPK_RS03945 | aerobactin synthase IucA | 2.972 | 1.571574 | 1.79E-15 |
| YPK_RS05995 | rseC | SoxR-reducing system protein RseC | 2.933 | 1.552166 | 6.63E-10 |
| YPK_RS11880 | tcyL | cystine ABC transporter permease | 2.923 | 1.547396 | 7.68E-21 |
| YPK_RS20830 | YPK_RS20830 | sulfate ABC transporter substrate-binding protein | 2.88 | 1.526248 | 1.61E-28 |
| YPK_RS13800 | psaA | adhesin PsaA | 2.878 | 1.525217 | 3.76E-10 |
| YPK_RS13190 | YPK_RS13190 | ribosome modulation factor | 2.873 | 1.522617 | 1.15E-34 |
| YPK_RS17825 | YPK_RS17825 | DNA polymerase II | 2.872 | 1.521959 | 4.64E-26 |

| | | | | | |
|---|---|---|---|---|---|
| YPK_RS07980 | YPK_RS07980 | MFS transporter | 2.871 | 1.521619 | 9.65E-23 |
| YPK_RS16840 | raiA | ribosome-associated translation inhibitor RaiA | 2.842 | 1.507097 | 6.6E-13 |
| YPK_RS20410 | YPK_RS20410 | hypothetical protein | 2.832 | 1.501929 | 0.007431 |
| YPK_RS13805 | YPK_RS13805 | protein PsaF | 2.81 | 1.490795 | 6.49E-18 |
| YPK_RS14950 | YPK_RS14950 | hypothetical protein | 2.786 | 1.478346 | 3.31E-27 |
| YPK_RS10525 | YPK_RS10525 | PrkA family serine protein kinase | 2.736 | 1.452004 | 8.51E-61 |
| YPK_RS00335 | YPK_RS00335 | ABC transporter permease | 2.716 | 1.441643 | 1.46E-22 |
| YPK_RS05660 | YPK_RS05660 | acid shock protein | 2.713 | 1.439906 | 0.005658 |
| YPK_RS05990 | rseB | sigma-E factor regulatory protein RseB | 2.709 | 1.437732 | 7.87E-57 |
| YPK_RS10530 | YPK_RS10530 | YeaH/YhbH family protein | 2.678 | 1.421039 | 1.35E-20 |
| YPK_RS03105 | YPK_RS03105 | integrase family protein | 2.647 | 1.404435 | 1.61E-59 |
| YPK_RS09290 | sufB | Fe-S cluster assembly protein SufB | 2.598 | 1.37726 | 1.79E-62 |
| YPK_RS09020 | YPK_RS09020 | metal-dependent hydrolase | 2.581 | 1.367806 | 0.001465 |
| YPK_RS13525 | poxB | ubiquinone-dependent pyruvate dehydrogenase | 2.58 | 1.367612 | 3.93E-47 |
| YPK_RS15615 | YPK_RS15615 | baseplate assembly protein | 2.556 | 1.353611 | 0.019965 |
| YPK_RS11335 | YPK_RS11335 | MaoC family dehydratase | 2.555 | 1.353313 | 0.02064 |
| YPK_RS19895 | YPK_RS19895 | dienelactone hydrolase family protein | 2.534 | 1.341346 | 5.67E-22 |
| YPK_RS00545 | YPK_RS00545 | XRE family transcriptional regulator | 2.522 | 1.334675 | 2.82E-14 |
| YPK_RS15575 | YPK_RS15575 | DinI-like family protein | 2.518 | 1.3321 | 1.43E-28 |
| YPK_RS04090 | YPK_RS04090 | ABC transporter permease | 2.508 | 1.326278 | 1.5E-12 |
| YPK_RS11875 | yecC | L-cystine ABC transporter ATP-binding protein YecC | 2.497 | 1.320221 | 3.43E-32 |
| YPK_RS09970 | YPK_RS09970 | MFS transporter | 2.471 | 1.305344 | 9.26E-19 |
| YPK_RS18225 | YPK_RS18225 | metal-dependent hydrolase | 2.468 | 1.303598 | 9.89E-05 |
| YPK_RS07525 | YPK_RS07525 | hypothetical protein | 2.457 | 1.297177 | 0.014269 |
| YPK_RS20770 | YPK_RS20770 | aquaporin family protein | 2.449 | 1.292026 | 2.52E-62 |
| YPK_RS11015 | YPK_RS11015 | hypothetical protein | 2.428 | 1.279979 | 5.18E-24 |
| YPK_RS06440 | iscR | Fe-S cluster assembly transcriptional regulator IscR | 2.422 | 1.276164 | 3.2E-52 |
| YPK_RS12665 | YPK_RS12665 | IS200/IS605-like element IS1541B family transposase | 2.401 | 1.263832 | 6.48E-07 |
| YPK_RS00585 | uspB | universal stress protein UspB | 2.394 | 1.259361 | 0.000505 |
| YPK_RS10560 | YPK_RS10560 | SpoVR family protein | 2.389 | 1.256544 | 1.76E-29 |
| YPK_RS20775 | glpK | glycerol kinase GlpK | 2.357 | 1.237031 | 5.93E-79 |
| YPK_RS12720 | YPK_RS12720 | IucA/IucC family siderophore biosynthesis protein | 2.335 | 1.223138 | 1.22E-15 |
| YPK_RS03925 | YPK_RS03925 | TonB-dependent siderophore receptor | 2.31 | 1.207974 | 5.26E-12 |
| YPK_RS01255 | YPK_RS01255 | LysE family translocator | 2.308 | 1.206796 | 9.34E-10 |

| | | | | | |
|---|---|---|---|---|---|
| YPK_RS12560 | YPK_RS12560 | MFS transporter | 2.298 | 1.200161 | 0.001003 |
| YPK_RS09285 | sufA | Fe-S cluster assembly scaffold SufA | 2.293 | 1.197412 | 9.15E-59 |
| YPK_RS08445 | dinI | DNA damage-inducible protein I | 2.293 | 1.196983 | 1.5E-14 |
| YPK_RS02305 | msrQ | protein-methionine-sulfoxide reductase heme-binding subunit MsrQ | 2.281 | 1.189656 | 6.7E-23 |
| YPK_RS11100 | YPK_RS11100 | anthranilate synthase component I family protein | 2.279 | 1.188258 | 0.002382 |
| YPK_RS01145 | cobA | uroporphyrinogen-III C-methyltransferase | 2.263 | 1.178148 | 7.22E-12 |
| YPK_RS01250 | YPK_RS01250 | hydrolase | 2.261 | 1.176966 | 2.94E-23 |
| YPK_RS09075 | YPK_RS09075 | IS256 family transposase | 2.242 | 1.164962 | 0.021772 |
| YPK_RS11620 | YPK_RS11620 | phage portal protein | 2.218 | 1.149275 | 0.00533 |
| YPK_RS16605 | YPK_RS16605 | transcriptional regulator | 2.211 | 1.144464 | 1.38E-05 |
| YPK_RS14335 | YPK_RS14335 | ABC transporter substrate-binding protein | 2.207 | 1.141781 | 1.34E-35 |
| YPK_RS01340 | tusB | sulfurtransferase complex subunit TusB | 2.2 | 1.137319 | 2.5E-09 |
| YPK_RS09295 | sufC | Fe-S cluster assembly ATPase SufC | 2.198 | 1.136453 | 1.11E-47 |
| YPK_RS16965 | recA | recombinase RecA | 2.197 | 1.13538 | 9.85E-98 |
| YPK_RS18560 | nrdG | anaerobic ribonucleoside-triphosphate reductase-activating protein | 2.194 | 1.133597 | 0.013164 |
| YPK_RS00425 | YPK_RS00425 | ABC transporter substrate-binding protein | 2.183 | 1.12634 | 2.53E-38 |
| YPK_RS09720 | YPK_RS09720 | hypothetical protein | 2.178 | 1.122958 | 0.002006 |
| YPK_RS07305 | YPK_RS07305 | cytochrome b | 2.176 | 1.12154 | 6.07E-06 |
| YPK_RS03165 | YPK_RS03165 | hypothetical protein | 2.173 | 1.119973 | 0.002852 |
| YPK_RS02430 | YPK_RS02430 | ribonuclease Ba | 2.17 | 1.11748 | 9.9E-13 |
| YPK_RS03770 | YPK_RS03770 | non-ribosomal peptide synthase | 2.163 | 1.113042 | 7.08E-26 |
| YPK_RS16855 | YPK_RS16855 | 3-deoxy-7-phosphoheptulonate synthase | 2.159 | 1.110157 | 6.63E-39 |
| YPK_RS01560 | YPK_RS01560 | DUF494 domain-containing protein | 2.159 | 1.110606 | 4.12E-16 |
| YPK_RS18520 | argF | ornithine carbamoyltransferase | 2.149 | 1.103648 | 9.79E-07 |
| YPK_RS06725 | YPK_RS06725 | 4-aminobutyrate--2-oxoglutarate transaminase | 2.136 | 1.094831 | 0.017093 |
| YPK_RS14330 | YPK_RS14330 | ABC transporter permease | 2.135 | 1.094074 | 1.51E-14 |
| YPK_RS15010 | nagA | N-acetylglucosamine-6-phosphate deacetylase | 2.129 | 1.090244 | 1.42E-47 |
| YPK_RS08090 | YPK_RS08090 | transporter | 2.126 | 1.088263 | 6.74E-47 |
| YPK_RS11150 | astA | arginine N-succinyltransferase | 2.125 | 1.087764 | 5.14E-07 |
| YPK_RS06665 | YPK_RS06665 | hypothetical protein | 2.124 | 1.086999 | 2.53E-16 |
| YPK_RS04255 | tkt | transketolase | 2.122 | 1.085625 | 7E-113 |
| YPK_RS01875 | lysC | lysine-sensitive aspartokinase 3 | 2.122 | 1.085127 | 1.78E-20 |
| YPK_RS01275 | tauD | taurine dioxygenase | 2.109 | 1.076818 | 5.82E-12 |
| YPK_RS05985 | rseA | anti-sigma-E factor RseA | 2.105 | 1.073574 | 4.12E-54 |

| | | | | | |
|---|---|---|---|---|---|
| YPK_RS19020 | YPK_RS19020 | DUF3757 domain-containing protein | 2.102 | 1.071699 | 2.61E-05 |
| YPK_RS01570 | tsaC | L-threonylcarbamoyladenylate synthase type 1 TsaC | 2.099 | 1.069595 | 5.15E-23 |
| YPK_RS01740 | YPK_RS01740 | sigma D regulator | 2.097 | 1.068022 | 2.78E-27 |
| YPK_RS15685 | YPK_RS15685 | DUF4942 domain-containing protein | 2.081 | 1.057124 | 0.005719 |
| YPK_RS13855 | xylB | xylulokinase | 2.08 | 1.056333 | 0.008532 |
| YPK_RS19870 | rmuC | DNA recombination protein RmuC | 2.077 | 1.054235 | 1.44E-22 |
| YPK_RS03175 | YPK_RS03175 | hypothetical protein | 2.077 | 1.054502 | 0.004039 |
| YPK_RS02235 | YPK_RS02235 | NAD(P)-dependent oxidoreductase | 2.067 | 1.047396 | 9.67E-07 |
| YPK_RS20095 | glpB | glycerol-3-phosphate dehydrogenase subunit GlpB | 2.057 | 1.040276 | 0.003228 |
| YPK_RS01565 | YPK_RS01565 | DNA topoisomerase | 2.051 | 1.036647 | 3.93E-24 |
| YPK_RS18800 | ftsH | ATP-dependent zinc metalloprotease FtsH | 2.05 | 1.035974 | 2.8E-102 |
| YPK_RS00080 | YPK_RS00080 | alpha-amylase | 2.046 | 1.033077 | 5.38E-07 |
| YPK_RS21120 | dtd | D-tyrosyl-tRNA(Tyr) deacylase | 2.041 | 1.029191 | 0.009863 |
| YPK_RS02730 | YPK_RS02730 | membrane protein | 2.038 | 1.02693 | 2.52E-21 |
| YPK_RS06935 | YPK_RS06935 | anaerobic sulfatase maturase | 2.023 | 1.016706 | 1.79E-09 |
| YPK_RS18210 | deoA | thymidine phosphorylase | 2.012 | 1.008979 | 2.51E-82 |
| YPK_RS11290 | YPK_RS11290 | PLP-dependent aminotransferase family protein | 2.011 | 1.00824 | 1.69E-14 |
| YPK_RS03050 | YPK_RS03050 | monooxygenase | 2.004 | 1.002983 | 5.67E-06 |
| YPK_RS18420 | YPK_RS18420 | DUF898 domain-containing protein | 0.5 | -1.0004 | 7.04E-05 |
| YPK_RS07890 | nuoE | NADH-quinone oxidoreductase subunit NuoE | 0.499 | -1.00208 | 1.75E-37 |
| YPK_RS05505 | proS | proline--tRNA ligase | 0.496 | -1.01117 | 4.14E-36 |
| YPK_RS21135 | typA | ribosome-dependent GTPase TypA | 0.491 | -1.0258 | 1.41E-85 |
| YPK_RS16875 | rplS | 50S ribosomal protein L19 | 0.489 | -1.03188 | 7.12E-57 |
| YPK_RS08540 | YPK_RS08540 | metal-dependent hydrolase | 0.489 | -1.03331 | 4.41E-14 |
| YPK_RS02340 | YPK_RS02340 | septum formation inhibitor Maf | 0.489 | -1.03166 | 1.69E-10 |
| YPK_RS19030 | rpsR | 30S ribosomal protein S18 | 0.488 | -1.03411 | 4.62E-13 |
| YPK_RS10090 | rsxC | electron transport complex subunit RsxC | 0.487 | -1.03716 | 0.000498 |
| YPK_RS16560 | nqrE | NADH:ubiquinone reductase (Na(+)-transporting) subunit E | 0.486 | -1.04019 | 9.21E-20 |
| YPK_RS16860 | yehT | two-component system response regulator BtsR | 0.486 | -1.04111 | 2.43E-08 |
| YPK_RS19010 | YPK_RS19010 | peptidyl-prolyl cis-trans isomerase | 0.485 | -1.04472 | 2.32E-76 |
| YPK_RS09165 | pheS | phenylalanine--tRNA ligase subunit alpha | 0.485 | -1.04528 | 9.83E-49 |
| YPK_RS13355 | rpsA | 30S ribosomal protein S1 | 0.484 | -1.0477 | 5.9E-114 |
| YPK_RS17460 | YPK_RS17460 | ABC transporter permease | 0.484 | -1.04627 | 3.14E-24 |
| YPK_RS16880 | trmD | tRNA (guanosine(37)-N1)-methyltransferase TrmD | 0.483 | -1.04871 | 5.5E-99 |

| | | | | | |
|---|---|---|---|---|---|
| YPK_RS04350 | gcvP | aminomethyl-transferring glycine dehydrogenase | 0.482 | -1.05225 | 2.81E-71 |
| YPK_RS19035 | priB | primosomal replication protein N | 0.481 | -1.0549 | 9.7E-51 |
| YPK_RS07905 | nuoH | NADH-quinone oxidoreductase subunit NuoH | 0.481 | -1.05684 | 2.01E-36 |
| YPK_RS16395 | proY | proline-specific permease ProY | 0.481 | -1.05485 | 1.87E-30 |
| YPK_RS07815 | YPK_RS07815 | phosphate acetyltransferase | 0.48 | -1.05891 | 1.85E-42 |
| YPK_RS04345 | gcvH | glycine cleavage system protein GcvH | 0.479 | -1.0621 | 1.04E-33 |
| YPK_RS01170 | tsgA | MFS transporter TsgA | 0.474 | -1.07757 | 4.56E-80 |
| YPK_RS18645 | YPK_RS18645 | sugar ABC transporter substrate-binding protein | 0.474 | -1.07581 | 1.93E-07 |
| YPK_RS08820 | YPK_RS08820 | hypothetical protein | 0.473 | -1.08046 | 1.42E-09 |
| YPK_RS18685 | YPK_RS18685 | U32 family peptidase | 0.473 | -1.07982 | 1.72E-06 |
| YPK_RS04960 | nagA | N-acetylglucosamine-6-phosphate deacetylase | 0.473 | -1.07896 | 0.000175 |
| YPK_RS16345 | secD | protein translocase subunit SecD | 0.472 | -1.08178 | 2.08E-68 |
| YPK_RS17010 | YPK_RS17010 | sugar ABC transporter ATP-binding protein | 0.471 | -1.08767 | 4.23E-05 |
| YPK_RS07885 | nuoC | NADH-quinone oxidoreductase subunit C/D | 0.47 | -1.08851 | 3.19E-68 |
| YPK_RS20555 | btuB | TonB-dependent vitamin B12 receptor BtuB | 0.47 | -1.0891 | 3.22E-21 |
| YPK_RS22730 | yidD | membrane protein insertion efficiency factor YidD | 0.469 | -1.09189 | 0.000912 |
| YPK_RS15825 | YPK_RS15825 | ABC transporter permease | 0.467 | -1.09926 | 1.01E-11 |
| YPK_RS16665 | YPK_RS16665 | amidohydrolase | 0.467 | -1.09885 | 7.43E-05 |
| YPK_RS10305 | ompW | outer membrane protein OmpW | 0.467 | -1.09814 | 0.000888 |
| YPK_RS07325 | YPK_RS07325 | lipoprotein | 0.466 | -1.10008 | 1.87E-08 |
| YPK_RS07320 | YPK_RS07320 | DUF4810 domain-containing protein | 0.466 | -1.10153 | 0.013527 |
| YPK_RS01355 | fusA | elongation factor G | 0.464 | -1.10672 | 7.9E-126 |
| YPK_RS01385 | rplC | 50S ribosomal protein L3 | 0.464 | -1.10764 | 1.1E-101 |
| YPK_RS15980 | rfbH | lipopolysaccharide biosynthesis protein RfbH | 0.464 | -1.10786 | 7.8E-70 |
| YPK_RS11265 | YPK_RS11265 | FTR1 family iron permease | 0.464 | -1.10882 | 2.14E-07 |
| YPK_RS18650 | YPK_RS18650 | alginate lyase family protein | 0.463 | -1.11046 | 1.26E-09 |
| YPK_RS06550 | der | ribosome biogenesis GTPase Der | 0.462 | -1.11431 | 2.28E-48 |
| YPK_RS09485 | dtpA | dipeptide/tripeptide permease DtpA | 0.462 | -1.11456 | 1.62E-25 |
| YPK_RS19025 | rplI | 50S ribosomal protein L9 | 0.461 | -1.11606 | 2.38E-92 |
| YPK_RS10335 | tonB | TonB system transport protein TonB | 0.46 | -1.11956 | 7.37E-06 |
| YPK_RS18070 | satP | acetate uptake transporter | 0.459 | -1.12418 | 2.56E-16 |
| YPK_RS01830 | metA | homoserine O-succinyltransferase | 0.459 | -1.12338 | 0.000648 |
| YPK_RS08265 | YPK_RS08265 | glycosidase | 0.458 | -1.12589 | 0.001452 |
| YPK_RS07805 | cmtB | PTS sugar transporter subunit IIA | 0.457 | -1.13017 | 2.27E-21 |

| | | | | | |
|---|---|---|---|---|---|
| YPK_RS04850 | YPK_RS04850 | sugar ABC transporter permease | 0.456 | -1.13159 | 0.000706 |
| YPK_RS09170 | pheT | phenylalanine--tRNA ligase subunit beta | 0.455 | -1.13495 | 5.1E-84 |
| YPK_RS06580 | guaA | glutamine-hydrolyzing GMP synthase | 0.455 | -1.13676 | 9.34E-56 |
| YPK_RS08665 | hflD | high frequency lysogenization protein HflD | 0.453 | -1.14128 | 5.72E-23 |
| YPK_RS13285 | mukE | chromosome partition protein MukE | 0.453 | -1.14279 | 7.15E-21 |
| YPK_RS19320 | rhaR | HTH-type transcriptional activator RhaR | 0.453 | -1.1432 | 8.13E-16 |
| YPK_RS15155 | mrdB | peptidoglycan glycosyltransferase MrdB | 0.452 | -1.14678 | 1.48E-12 |
| YPK_RS09055 | kduD | 2-dehydro-3-deoxy-D-gluconate 5-dehydrogenase KduD | 0.451 | -1.14726 | 6.8E-20 |
| YPK_RS21065 | YPK_RS21065 | trimeric intracellular cation channel family protein | 0.451 | -1.14873 | 4.37E-12 |
| YPK_RS21355 | glmS | glutamine--fructose-6-phosphate transaminase (isomerizing) | 0.45 | -1.15279 | 1.1E-99 |
| YPK_RS15990 | rfbF | glucose-1-phosphate cytidylyltransferase | 0.448 | -1.15961 | 8.27E-44 |
| YPK_RS04005 | tssC | type VI secretion system contractile sheath large subunit | 0.446 | -1.16353 | 0.002171 |
| YPK_RS18000 | carB | carbamoyl-phosphate synthase large subunit | 0.445 | -1.16962 | 4.1E-99 |
| YPK_RS06800 | purM | phosphoribosylformylglycinamidine cyclo-ligase | 0.443 | -1.17456 | 1.74E-59 |
| YPK_RS02270 | dusB | tRNA dihydrouridine synthase DusB | 0.443 | -1.17551 | 1.71E-40 |
| YPK_RS15130 | holA | DNA polymerase III subunit delta | 0.443 | -1.17515 | 6.76E-22 |
| YPK_RS00230 | YPK_RS00230 | MFS transporter | 0.443 | -1.17544 | 1.45E-20 |
| YPK_RS06810 | uraA | uracil permease | 0.443 | -1.17568 | 1.02E-11 |
| YPK_RS06570 | guaB | IMP dehydrogenase | 0.44 | -1.18382 | 7.8E-53 |
| YPK_RS20995 | pyrE | orotate phosphoribosyltransferase | 0.439 | -1.18816 | 1.64E-34 |
| YPK_RS06795 | purN | phosphoribosylglycinamide formyltransferase | 0.439 | -1.18638 | 1.15E-06 |
| YPK_RS14990 | glnS | glutamine--tRNA ligase | 0.438 | -1.19247 | 1.6E-111 |
| YPK_RS04985 | YPK_RS04985 | SIS domain-containing protein | 0.438 | -1.1915 | 3.06E-09 |
| YPK_RS16430 | YPK_RS16430 | AAA family ATPase | 0.437 | -1.19317 | 5.24E-42 |
| YPK_RS16300 | pgpA | phosphatidylglycerophosphatase A | 0.437 | -1.19322 | 3.21E-23 |
| YPK_RS00055 | YPK_RS00055 | heat shock protein IbpA | 0.437 | -1.19482 | 1.25E-19 |
| YPK_RS19040 | rpsF | 30S ribosomal protein S6 | 0.436 | -1.19795 | 8.28E-88 |
| YPK_RS13100 | rlmI | 23S rRNA (cytosine(1962)-C(5))-methyltransferase RlmI | 0.436 | -1.19732 | 4.43E-34 |
| YPK_RS21300 | rsmG | 16S rRNA (guanine(527)-N(7))-methyltransferase RsmG | 0.432 | -1.21023 | 4.72E-24 |
| YPK_RS12230 | purT | formate-dependent phosphoribosylglycinamide formyltransferase | 0.432 | -1.21033 | 1.08E-13 |
| YPK_RS00475 | dctA | dicarboxylate/amino acid:cation symporter | 0.429 | -1.22122 | 1.49E-78 |
| YPK_RS13760 | YPK_RS13760 | iron ABC transporter permease | 0.429 | -1.221 | 9.57E-24 |
| YPK_RS23245 | hemP | hemin uptake protein HemP | 0.428 | -1.22466 | 0.004846 |
| YPK_RS16660 | YPK_RS16660 | pyridoxal phosphate-dependent aminotransferase | 0.427 | -1.2282 | 3.32E-12 |

| | | | | | |
|---|---|---|---|---|---|
| YPK_RS19210 | frdD | fumarate reductase subunit FrdD | 0.425 | -1.23563 | 1.84E-17 |
| YPK_RS15985 | rfbG | CDP-glucose 4%2C6-dehydratase | 0.424 | -1.23875 | 1E-50 |
| YPK_RS12495 | YPK_RS12495 | response regulator transcription factor | 0.424 | -1.23858 | 5.46E-10 |
| YPK_RS05525 | YPK_RS05525 | D-methionine ABC transporter permease MetI | 0.423 | -1.24265 | 1.59E-12 |
| YPK_RS20660 | metF | methylenetetrahydrofolate reductase | 0.422 | -1.24352 | 3.55E-05 |
| YPK_RS01470 | rpsE | 30S ribosomal protein S5 | 0.421 | -1.24661 | 6.12E-90 |
| YPK_RS00115 | glyS | glycine--tRNA ligase subunit beta | 0.42 | -1.25169 | 8.63E-78 |
| YPK_RS12145 | YPK_RS12145 | flagellar protein FlhE | 0.42 | -1.25165 | 5.2E-17 |
| YPK_RS23200 | YPK_RS23200 | hypothetical protein | 0.419 | -1.25406 | 8.57E-06 |
| YPK_RS18505 | YPK_RS18505 | valine--tRNA ligase | 0.418 | -1.25939 | 1.83E-46 |
| YPK_RS13570 | artM | arginine ABC transporter permease ArtM | 0.417 | -1.26047 | 0.000411 |
| YPK_RS01445 | rplE | 50S ribosomal protein L5 | 0.416 | -1.26388 | 1.2E-134 |
| YPK_RS16580 | YPK_RS16580 | Na(+)-translocating NADH-quinone reductase subunit A | 0.416 | -1.26587 | 1.14E-81 |
| YPK_RS21105 | YPK_RS21105 | uracil-xanthine permease | 0.415 | -1.26782 | 7.43E-08 |
| YPK_RS01380 | rpsJ | 30S ribosomal protein S10 | 0.411 | -1.28395 | 1.7E-133 |
| YPK_RS21410 | YPK_RS21410 | NCS2 family permease | 0.411 | -1.28138 | 7.32E-14 |
| YPK_RS13755 | YPK_RS13755 | ABC transporter ATP-binding protein | 0.409 | -1.29048 | 4.8E-14 |
| YPK_RS04970 | YPK_RS04970 | PTS mannose/fructose/sorbose transporter family subunit IID | 0.408 | -1.29212 | 2.94E-15 |
| YPK_RS12960 | YPK_RS12960 | type VI secretion system membrane subunit | 0.407 | -1.29809 | 3.63E-08 |
| YPK_RS10935 | prs | ribose-phosphate pyrophosphokinase | 0.405 | -1.30363 | 4.9E-125 |
| YPK_RS08015 | menF | isochorismate synthase MenF | 0.405 | -1.30225 | 4.38E-14 |
| YPK_RS07745 | purF | amidophosphoribosyltransferase | 0.402 | -1.31429 | 1.19E-60 |
| YPK_RS13565 | artQ | arginine ABC transporter permease ArtQ | 0.4 | -1.32048 | 2.58E-05 |
| YPK_RS13215 | YPK_RS13215 | ABC transporter ATP-binding protein | 0.399 | -1.32375 | 6.78E-93 |
| YPK_RS04940 | YPK_RS04940 | sulfatase-like hydrolase/transferase | 0.398 | -1.32842 | 6.59E-15 |
| YPK_RS02460 | YPK_RS02460 | nucleoside diphosphate kinase regulator | 0.397 | -1.33111 | 3.87E-60 |
| YPK_RS13560 | YPK_RS13560 | arginine ABC transporter substrate-binding protein | 0.395 | -1.33926 | 1.67E-23 |
| YPK_RS01465 | rplR | 50S ribosomal protein L18 | 0.394 | -1.34346 | 3.5E-127 |
| YPK_RS06470 | fdx | ISC system 2Fe-2S type ferredoxin | 0.392 | -1.35166 | 5.74E-12 |
| YPK_RS14845 | YPK_RS14845 | cytochrome d ubiquinol oxidase subunit II | 0.39 | -1.35994 | 2.02E-78 |
| YPK_RS15355 | YPK_RS15355 | cupin domain-containing protein | 0.389 | -1.36268 | 7.76E-05 |
| YPK_RS04975 | YPK_RS04975 | PTS mannose/fructose/sorbose/N-acetylgalactosamine transporter subuni | 0.387 | -1.36782 | 7.35E-13 |
| YPK_RS18220 | YPK_RS18220 | NupC/NupG family nucleoside CNT transporter | 0.386 | -1.3728 | 3.23E-59 |
| YPK_RS12340 | YPK_RS12340 | TonB-dependent receptor | 0.385 | -1.37594 | 4.14E-53 |

| | | | | | |
|---|---|---|---|---|---|
| YPK_RS00660 | YPK_RS00660 | gluconokinase | 0.385 | -1.37755 | 2.05E-45 |
| YPK_RS16565 | YPK_RS16565 | NADH:ubiquinone reductase (Na(+)-transporting) subunit D | 0.382 | -1.38731 | 1.1E-38 |
| YPK_RS01460 | rplF | 50S ribosomal protein L6 | 0.377 | -1.40789 | 4.4E-131 |
| YPK_RS17385 | fhuB | Fe(3+)-hydroxamate ABC transporter permease FhuB | 0.376 | -1.41025 | 7.3E-28 |
| YPK_RS21100 | gltS | sodium/glutamate symporter | 0.375 | -1.41354 | 2.42E-96 |
| YPK_RS04630 | lysS | lysine--tRNA ligase | 0.371 | -1.43102 | 2.5E-149 |
| YPK_RS01450 | rpsN | 30S ribosomal protein S14 | 0.371 | -1.42923 | 2.15E-47 |
| YPK_RS06475 | iscX | Fe-S cluster assembly protein IscX | 0.371 | -1.42898 | 0.00726 |
| YPK_RS02335 | mreD | rod shape-determining protein MreD | 0.368 | -1.44399 | 1.34E-09 |
| YPK_RS12590 | YPK_RS12590 | hypothetical protein | 0.367 | -1.44478 | 1.01E-15 |
| YPK_RS05390 | pyrH | UMP kinase | 0.365 | -1.45274 | 5.6E-127 |
| YPK_RS16355 | tgt | tRNA guanosine(34) transglycosylase Tgt | 0.365 | -1.45485 | 6.66E-60 |
| YPK_RS13705 | YPK_RS13705 | HAAAP family serine/threonine permease | 0.361 | -1.46931 | 2.5E-112 |
| YPK_RS19200 | YPK_RS19200 | succinate dehydrogenase/fumarate reductase iron-sulfur subunit | 0.361 | -1.46845 | 5.58E-44 |
| YPK_RS05530 | metN | methionine ABC transporter ATP-binding protein MetN | 0.361 | -1.47131 | 3.92E-36 |
| YPK_RS14850 | cydA | cytochrome ubiquinol oxidase subunit I | 0.358 | -1.4802 | 8.7E-104 |
| YPK_RS09375 | sodB | superoxide dismutase [Fe] | 0.353 | -1.501 | 1.4E-21 |
| YPK_RS14495 | rhlE | ATP-dependent RNA helicase RhlE | 0.35 | -1.51398 | 2.33E-47 |
| YPK_RS18730 | YPK_RS18730 | DEAD/DEAH family ATP-dependent RNA helicase | 0.347 | -1.52571 | 2.32E-83 |
| YPK_RS15815 | purE | 5-(carboxyamino)imidazole ribonucleotide mutase | 0.347 | -1.52512 | 1.19E-08 |
| YPK_RS19205 | frdC | fumarate reductase subunit FrdC | 0.346 | -1.53266 | 4.12E-12 |
| YPK_RS09060 | kduI | 5-dehydro-4-deoxy-D-glucuronate isomerase | 0.345 | -1.53701 | 5.21E-14 |
| YPK_RS06965 | napF | ferredoxin-type protein NapF | 0.343 | -1.54317 | 1.69E-24 |
| YPK_RS02265 | fis | DNA-binding transcriptional regulator Fis | 0.342 | -1.54962 | 2E-105 |
| YPK_RS14170 | YPK_RS14170 | MFS transporter | 0.342 | -1.54618 | 3.81E-06 |
| YPK_RS13765 | YPK_RS13765 | ABC transporter substrate-binding protein | 0.341 | -1.55274 | 1.63E-55 |
| YPK_RS16750 | emrB | multidrug efflux MFS transporter permease subunit EmrB | 0.339 | -1.56147 | 1.21E-25 |
| YPK_RS01455 | rpsH | 30S ribosomal protein S8 | 0.336 | -1.57267 | 6.2E-144 |
| YPK_RS16575 | YPK_RS16575 | NADH:ubiquinone reductase (Na(+)-transporting) subunit B | 0.333 | -1.58591 | 1.7E-120 |
| YPK_RS00590 | pitA | inorganic phosphate transporter PitA | 0.329 | -1.60456 | 1.45E-96 |
| YPK_RS04955 | kduD | 2-dehydro-3-deoxy-D-gluconate 5-dehydrogenase KduD | 0.329 | -1.6035 | 2.93E-12 |
| YPK_RS21440 | yidC | membrane protein insertase YidC | 0.328 | -1.60628 | 4.48E-17 |
| YPK_RS01780 | purD | phosphoribosylamine--glycine ligase | 0.327 | -1.61116 | 1.15E-60 |
| YPK_RS19195 | frdA | fumarate reductase (quinol) flavoprotein subunit | 0.324 | -1.62394 | 2.2E-138 |

| | | | | | |
|---|---|---|---|---|---|
| YPK_RS08660 | purB | adenylosuccinate lyase | 0.322 | -1.63569 | 3.4E-99 |
| YPK_RS13390 | YPK_RS13390 | 30S ribosomal protein S12 methylthiotransferase accessory protein YcaO | 0.316 | -1.66392 | 1.24E-57 |
| YPK_RS16570 | YPK_RS16570 | Na(+)-translocating NADH-quinone reductase subunit C | 0.31 | -1.69001 | 2.1E-101 |
| YPK_RS04225 | metK | methionine adenosyltransferase | 0.308 | -1.69677 | 1.8E-153 |
| YPK_RS01785 | purH | bifunctional phosphoribosylaminoimidazolecarboxamide formyltransferase | 0.305 | -1.71125 | 2.98E-71 |
| YPK_RS16275 | thiI | tRNA 4-thiouridine(8) synthase ThiI | 0.305 | -1.71184 | 1.67E-50 |
| YPK_RS17830 | rapA | RNA polymerase-associated protein RapA | 0.301 | -1.73308 | 2.36E-70 |
| YPK_RS18330 | YPK_RS18330 | O-antigen ligase family protein | 0.294 | -1.76685 | 6.2E-105 |
| YPK_RS02130 | YPK_RS02130 | NCS2 family permease | 0.291 | -1.78326 | 2.12E-12 |
| YPK_RS06980 | napB | nitrate reductase cytochrome c-type subunit | 0.29 | -1.78362 | 3.25E-15 |
| YPK_RS19545 | YPK_RS19545 | heme anaerobic degradation radical SAM methyltransferase ChuW/HutW | 0.286 | -1.80421 | 3.39E-25 |
| YPK_RS06975 | napA | nitrate reductase catalytic subunit NapA | 0.278 | -1.84704 | 9.79E-23 |
| YPK_RS16340 | secF | protein translocase subunit SecF | 0.272 | -1.87903 | 8.3E-168 |
| YPK_RS06340 | purL | phosphoribosylformylglycinamidine synthase | 0.269 | -1.89263 | 7.4E-137 |
| YPK_RS15125 | lptE | LPS assembly lipoprotein LptE | 0.266 | -1.9082 | 1.68E-49 |
| YPK_RS05015 | YPK_RS05015 | hypothetical protein | 0.265 | -1.91524 | 4.12E-16 |
| YPK_RS08235 | ugpC | sn-glycerol-3-phosphate ABC transporter ATP-binding protein UgpC | 0.265 | -1.91441 | 2.04E-15 |
| YPK_RS04100 | YPK_RS04100 | ABC transporter substrate-binding protein | 0.258 | -1.95662 | 2.37E-39 |
| YPK_RS12585 | YPK_RS12585 | APC family permease | 0.254 | -1.9767 | 7.5E-162 |
| YPK_RS12350 | YPK_RS12350 | hypothetical protein | 0.251 | -1.99632 | 3.34E-09 |
| YPK_RS04980 | YPK_RS04980 | PTS system mannose/fructose/N-acetylgalactosamine-transporter subunit | 0.249 | -2.00757 | 6.05E-13 |
| YPK_RS06430 | suhB | inositol-1-monophosphatase | 0.228 | -2.13503 | 1.6E-116 |
| YPK_RS05010 | yapM | autotransporter YapM | 0.223 | -2.16361 | 2.16E-20 |
| YPK_RS17100 | YPK_RS17100 | fumarate hydratase | 0.211 | -2.24599 | 2.1E-191 |
| YPK_RS15820 | purK | 5-(carboxyamino)imidazole ribonucleotide synthase | 0.188 | -2.41276 | 4.83E-53 |
| YPK_RS04095 | YPK_RS04095 | TonB-dependent siderophore receptor | 0.16 | -2.64529 | 1.2E-122 |

Table S2 Operons under OxyR control in YPIII

| seq_id | gene | function | strand | start | end | sequence | weight |
|---|---|---|---|---|---|---|---|
| YPK_RS17025 | katG | catalase/peroxidase HPI | D | -99 | -61 | AATAGATAGAATCTATCATATTAATAGGACTAACGAATT | 23.1 |
| YPK_RS14285 | katE | catalase | D | -192 | -154 | AATAGCGATTACCCATGTTAACAATAAGGCATACCTATT | 22.7 |
| YPK_RS16370 | ahpC | peroxiredoxin C | D | -123 | -85 | GATAGGCAAAAACTATCATAACAATTGGTATTTATTCCT | 21.1 |
| YPK_RS13445 | trxB | thioredoxin-disulfide reductase | D | -113 | -75 | AATAGGCTTTACCTATCAAAAAAATTGCTAACAACATTT | 21.0 |
| YPK_RS13675 | grxA | GrxA family glutaredoxin | D | -170 | -132 | AATAGATAAAATTAATCAAACTAACAGGTAATTCCTGCT | 12.6 |
| YPK_RS08095 | dps | DNA starvation/stationary phase protection protein Dps | D | -92 | -54 | GATAGGCAGCATCTGACCTGCAAAATAAAATACCTTTGT | 12.4 |
| YPK_RS16760 | trxC | thioredoxin TrxC | D | -181 | -143 | AAAAGCTACCAACTATCGTACTCATCGAATAAACCATTT | 12.1 |
| YPK_RS13765 | - | iron-siderophore ABC transporter substrate-binding protein YiuA | R | -85 | -47 | AAATGATAATACTTATCAATAAGATTGCCATAAGTAATT | 11.6 |
| YPK_RS08125 | - | hypothetical protein | R | -80 | -42 | AATAAGGACGCTCAATGTTGATAAGGAGTATTAATTATT | 10.7 |
| YPK_RS14005 | - | hypothetical protein | R | -141 | -103 | AAGAGTGAAAATCAATTAAGTAGATAATTTAAGACTTTA | 10.3 |
| YPK_RS07130 | - | pyridoxal phosphate-dependent aminotransferase | D | -68 | -30 | AATAGTAAGAAATTATCAAGATAATGGTATTAAATTTCC | 10.1 |
| YPK_RS00340 | - | siderophore ABC transporter substrate-binding protein | R | -66 | -28 | AATCAGCATCAGTAATGCTAATGATTTTTATTACCAATC | 9.8 |
| YPK_RS03285 | ygiD | 4,5-DOPA dioxygenase extradiol | R | -278 | -240 | GATAATAAGTAGCAATGGTAATGAATAGCAACGACAACC | 9.8 |
| YPK_RS11100 | - | anthranilate synthase component I family protein | D | -71 | -33 | GATAGAAATTATCATTAAAATAAATACAAAAAAATAATT | 9.5 |
| YPK_RS10200 | acnA | aconitate hydratase AcnA | D | -292 | -254 | CTTAGGTGATACCTATCACTATGAATGCTCACCATTGTT | 9.1 |
| YPK_RS17920 | - | ImpA family type VI secretion system protein | D | -191 | -153 | AATAAGAAAAAAATAAAGACATAATAGCTATAACCTATC | 8.8 |
| YPK_RS10680 | zwf | glucose-6-phosphate dehydrogenase | R | -227 | -189 | GATAACCATTTACCATTATGAAAATTAGACACAGGTCAT | 8.7 |
| YPK_RS10685 | hexR | MurR/RpiR family transcriptional regulator | D | -173 | -135 | GATAACCATTTACCATTATGAAAATTAGACACAGGTCAT | 8.7 |
| YPK_RS17135 | - | heme-binding domain-containing protein | D | -198 | -160 | CATAGGCAATACCTATTCCAGCAATAGGCAAAGCCGCCC | 8.7 |

| | | | | | | | |
|---|---|---|---|---|---|---|---|
| YPK RS09520 | - | hypothetical protein | D | -73 | -35 | AAATGATAATACTTATCAATATCAATTGTAAATAGATTT | 8.6 |
| YPK_RS00455 | hmsP | biofilm formation regulator HmsP | D | -136 | -98 | GGTAGGGCGGATCTGGCCTAATGATAGGGAAATGCGCTT | 8.3 |
| YPK_RS10560 | - | SpoVR family protein | D | -118 | -80 | GGAAGAAATAATCTATATTGACATAAGGTCTTGCCATGC | 8.3 |
| YPK_RS19400 | pspG | envelope stress response protein PspG | R | -265 | -227 | AATTCATTGAACTTATTGTAAAAATAATAAATAGCTAAC | 8.0 |
| YPK_RS05680 | cspA | RNA chaperone/antiterminator CspA | R | -244 | -206 | AATAGCAAGACTTAATTATTACACTTGCGAATAGGTAAC | 7.9 |
| YPK_RS03395 | exbB | tol-pal system-associated acyl-CoA thioesterase | R | -87 | -49 | GATTGATAGTAATTATCATTAGTATTTGGCAAGATTTTT | 7.8 |
| YPK_RS12250 | - | L-serine ammonia-lyase | D | -292 | -254 | AATAACTAGAACGTATTTTGTTAAATAGGGATAATTTTC | 7.8 |
| YPK_RS01875 | lysC | lysine-sensitive aspartokinase 3 | R | -283 | -245 | GAAACAGATCAAGCGTTGTTTCGAGAGATAATACCTATT | 7.6 |
| YPK RS01880 | - | glucose-6-phosphate isomerase | D | -125 | -87 | GAAACAGATCAAGCGTTGTTTCGAGAGATAATACCTATT | 7.6 |
| YPK_RS03645 | - | transcriptional regulator | D | -225 | -187 | AATAATACATAGCAATCGAGAAGAATAGTTAACCATCCT | 7.5 |
| YPK_RS08775 | - | MgtC family protein | D | -56 | -18 | AACTCGAATTATTTAGGGTATAAATAGCACATGATTATT | 7.5 |
| YPK_RS05100 | - | type II secretion system pilot lipoprotein GspS | D | -233 | -195 | ACAAAGAAAAACTAATTGTAATTATTAATAAAACGATTT | 7.4 |
| YPK_RS05105 | - | hypothetical protein | R | -89 | -51 | ACAAAGAAAAACTAATTGTAATTATTAATAAAACGATTT | 7.4 |
| YPK_RS10415 | - | DNA-binding transcriptional regulator H-NS | D | -81 | -43 | AATTGGCTATTGCAGAAATTAAAATAGCACTCTATTATT | 7.4 |
| YPK_RS13800 | psaA | adhesin PsaA | R | -42 | -4 | AACAGTCTCCATTAAATGTAATAATTGCTATTACGACAT | 7.4 |
| YPK_RS11790 | uvrY | UvrY/SirA/GacA family response regulator transcription factor | D | -283 | -245 | CATAGGAATTAACTTATCTAATGCTAATAATTAGTTTTT | 7.2 |
| YPK_RS11810 | - | Ail/Lom family outer membrane beta-barrel protein | R | -230 | -192 | AATAACAACAAGCATTAGTATAAATTAGGCTTGGTTATT | 7.2 |
| YPK RS03035 | - | histidine kinase | D | -135 | -97 | AATAAAATGTTATTATGATAATAAATAATATCGCCATTT | 7.1 |
| YPK RS07265 | mntH | Nramp family divalent metal transporter | R | -104 | -66 | AATTATCAAGCGCATTTGTGATAATGGAATTATCTTTTT | 7.1 |
